# Supplementary material for: Electrocatalytic on-site oxygenation for transplanted cell-based-therapies
Source: Nat Commun. 2023 Nov 9;14:7019. doi: 10.1038/s41467-023-42697-2 (PMC10636048; doi:10.1038/s41467-023-42697-2)
Supplement: Supplementary file 1 — Supplementary Information [file 41467_2023_42697_MOESM1_ESM.pdf]

Supplementary Information for:

Electrocatalytic on-site oxygenation for transplanted cell-based-therapies

Inkyu Lee[1†], Abhijith Surendran[2†], Samantha Fleury[3], Ian Gimino[4], Alexander Curtiss[5], Cody Fell[3], Daniel J. Shiwarski[6], Omar Refy[7], Blaine Rothrock[8], Seonghan Jo[1], Tim Schwartzkopff[4], Abijeet Singh Mehta[2], Yingqiao Wang[1], Adam Sipe[9], Sharon John[10], Xudong Ji[2,11], Georgios Nikiforidis[2], Adam W. Feinberg[1,6], Josiah Hester[12], Douglas J. Weber[6,10,13], Omid Veiseh[3], Jonathan Rivnay[2,11,14]*, and Tzahi Cohen-Karni[1,6]*

[1] Department of Materials Science and Engineering, Carnegie Mellon University, Pittsburgh, PA, U.S.A.

[2] Department of Biomedical Engineering, Northwestern University, Evanston, IL, U.S.A.

[3] Department of Bioengineering, Rice University, Houston, TX, U.S.A.

[4] Department of Chemical Engineering, Carnegie Mellon University, Pittsburgh, PA, U.S.A.

[5] Department of Electrical and Computer Engineering, Northwestern University, IL, U.S.A.

[6] Department of Biomedical Engineering, Carnegie Mellon University, Pittsburgh, PA, U.S.A.

[7] Department of Physics, Carnegie Mellon University, Pittsburgh, PA, U.S.A.

[8] Department of Computer Science, Northwestern University, Evanston, IL, U.S.A.

[9] Department of Material Science and Engineering, The Pennsylvania State University, State College, PA, U.S.A.

[10] Neuroscience Institute, Carnegie Mellon University, Pittsburgh, PA, U.S.A.

[11] Simpson Querrey Institute, Northwestern University, Chicago, IL, U.S.A.

[12] Interactive Computing and Computer Science, Georgia Institute of Technology, Atlanta, GA, U.S.A.

[13] Department of Mechanical Engineering, Carnegie Mellon University, Pittsburgh, PA, U.S.A.

[14] Department of Materials Science and Engineering, Northwestern University, Evanston, IL, 60208 USA.

* Tzahi Cohen-Karni: tzahi@andrew.cmu.edu / Jonathan Rivnay: jrivnay@northwestern.edu

Supplementary Tables

Supplementary Table 1. Data summary of XPS elemental analysis. Samples were prepared on Si/SiO_2 (600 nm) wafer. Given trace level of Si signal, collected atomic fractions were free from the substrates. Results are presented as mean ± SD (*n*=4).

	**Element**	**Atomic ratio (%)**
**Sample #1**	Ir	23.6
	O	64.3
	C	12.1
	Si	trace (< 0.5)
**Sample #2**	Ir	22.5
	O	63.9
	C	13.6
	Si	trace (< 0.5)
**Sample #3**	Ir	23.4
	O	65.8
	C	10.9
	Si	trace (< 0.5)
**Sample #4**	Ir	21.7
	O	62.9
	C	15.4
	Si	trace (< 0.5)
**Average**	Ir	22.8 ± 0.76
	O	64.23 ± 1.04
	C	13.0 ± 1.69
	Si	trace (< 0.5)

Supplementary Table 2. Data summary for XPS of SIROF in C 1s region. C-C state at 284.4 eV in C 1s region was utilized for chemical shift calibration. Results are presented as mean ± SD (*n*=4).

**Peak**	**Sample**	**State**	**Peak BE** (eV)	**FWHM** (eV)	**Ratio**
**C 1s**	**Sample #1**	C-C	284.8	1.7	100
	**Sample #2**	C-C	284.8	1.8	100
	**Sample #3**	C-C	284.8	1.8	100
	**Sample #4**	C-C	284.8	1.7	100
	**Average**	C-C	284.8 ± 0	1.8 ± 0.05	100 ± 0

Supplementary Table 3. Data summary for XPS of SIROF in Ir 4f region. Results are presented as mean ± SD (n=4).

**Peak**	**Sample**	**State**	**Peak BE** (eV)	**FWHM** (eV)	**Ratio**
**Ir 4f**[8]	**Sample #1**	Ir(IV) 7/2	62.2	1.8	39.6
		Ir(IV) 5/2	65.2	1.8	39.8
		Ir (IV) 7/2 sat.	63.4	2.9	10.3
		Ir (IV) 5/2 sat.	66.6	2.9	10.4
	**Sample #2**	Ir(IV) 7/2	62.2	1.8	38.5
		Ir(IV) 5/2	65.2	1.8	38.7
		Ir (IV) 7/2 sat.	63.4	3.0	11.4
		Ir (IV) 5/2 sat.	66.6	3.0	11.4
	**Sample #3**	Ir(IV) 7/2	62.3	1.8	38.3
		Ir(IV) 5/2	65.3	1.8	38.3
		Ir (IV) 7/2 sat.	63.4	2.9	11.7
		Ir (IV) 5/2 sat.	66.6	2.9	11.7
	**Sample #4**	Ir(IV) 7/2	62.1	1.8	38.7
		Ir(IV) 5/2	65.1	1.8	38.7
		Ir (IV) 7/2 sat.	63.2	3.0	11.3
		Ir (IV) 5/2 sat.	66.4	3.0	11.3
	**Average**	Ir(IV) 7/2	62.2 ± 0.07	1.8 ± 0	38.8 ± 0.50
		Ir(IV) 5/2	65.2 ± 0.07	1.8 ± 0	38.8 ± 0.56
		Ir (IV) 7/2 sat.	63.4 ± 0.09	3.0 ± 0.05	11.2 ± 0.53
		Ir (IV) 5/2 sat.	66.6 ±0.09	3.0 ± 0.05	11.2 ± 0.49

Supplementary Table 4. Data summary for XPS of SIROF in O 1s region. Results are presented as mean ± SD (*n*=4).

**Peak**	**Sample**	**State**	**Peak BE** (eV)	**FWHM** (eV)	**Ratio**
**O 1s**[10,11]	**Sample #1**	IrO_2 (lattice)	530.5	1.5	40.8
		IrO_X (vacancy)	531.6	1.5	29.7
		IrOH	532.6	1.5	18.5
		C-O	533.7	1.5	8.4
		H_2O, abs.	535.1	1.5	2.7
	**Sample #2**	IrO_2 (lattice)	530.6	1.4	40.3
		IrO_X (vacancy)	531.7	1.4	30.7
		IrOH	532.7	1.4	18.7
		C-O	533.7	1.4	7.9
		H_2O, abs.	535.0	1.4	2.3
	**Sample #3**	IrO_2 (lattice)	530.5	1.5	38.2
		IrO_X (vacancy)	531.6	1.5	27.4
		IrOH	532.5	1.5	15.0
		C-O	533.5	1.5	8.8
		H_2O, abs.	534.9	1.5	3.0
	**Sample #4**	IrO_2 (lattice)	530.5	1.5	41.7
		IrO_X (vacancy)	531.6	1.5	32.1
		IrOH	532.6	1.5	16.0
		C-O	533.8	1.5	7.8
		H_2O, abs.	535.3	1.5	2.4
	**Average**	IrO_2 (lattice)	530.5 ± 0.04	1.5 ± 0.04	40.3 ± 1.29
		IrO_X (vacancy)	531.6 ± 0.04	1.5 ± 0.04	30.0 ± 1.71
		IrOH	532.6 ± 0.07	1.5 ± 0.04	17.1 ± 1.59
		C-O	533.7 ± 0.11	1.5 ± 0.04	8.2 ± 0.40
		H_2O, abs.	535.1 ± 0.15	1.5 ± 0.04	2.6 ± 0.27

Supplementary Figures

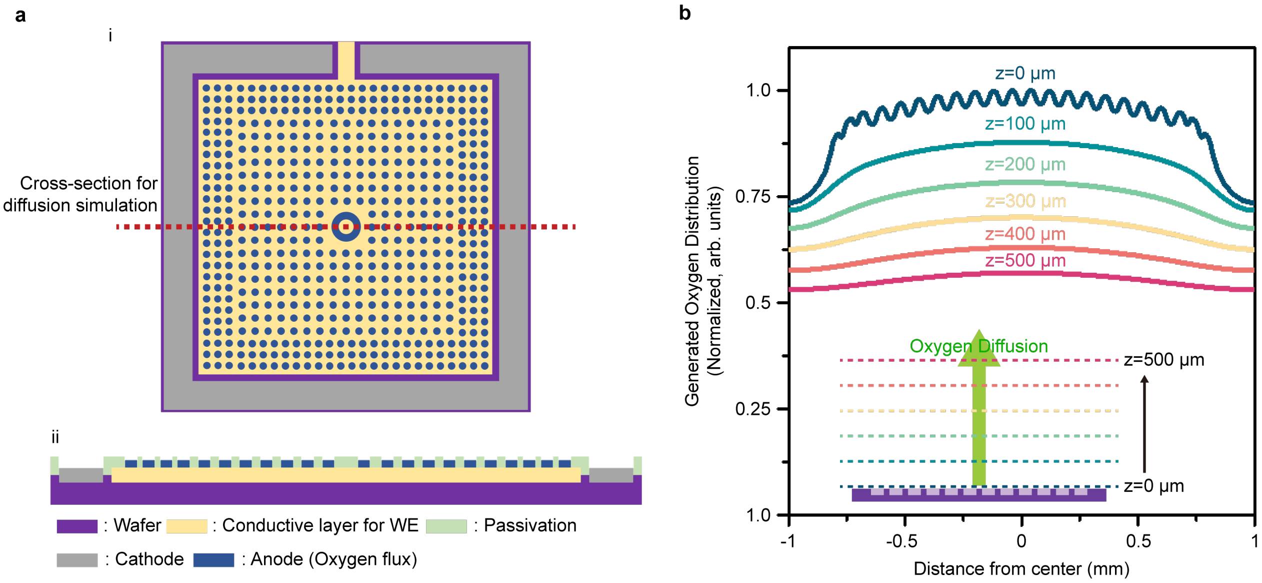

Supplementary Figure 1. Simulated cross-sectional oxygen profiles. (a) The designed electrode for diffusion simulation; i – top view of the electrode; ii – cross-sectional view of the electrode at the center of the electrode. (b) The simulated oxygen profiles at different z-positions. The oxygen levels were normalized with the value at z=0 µm; inset: a schematic illustration describing how the profiles were exported and plotted.

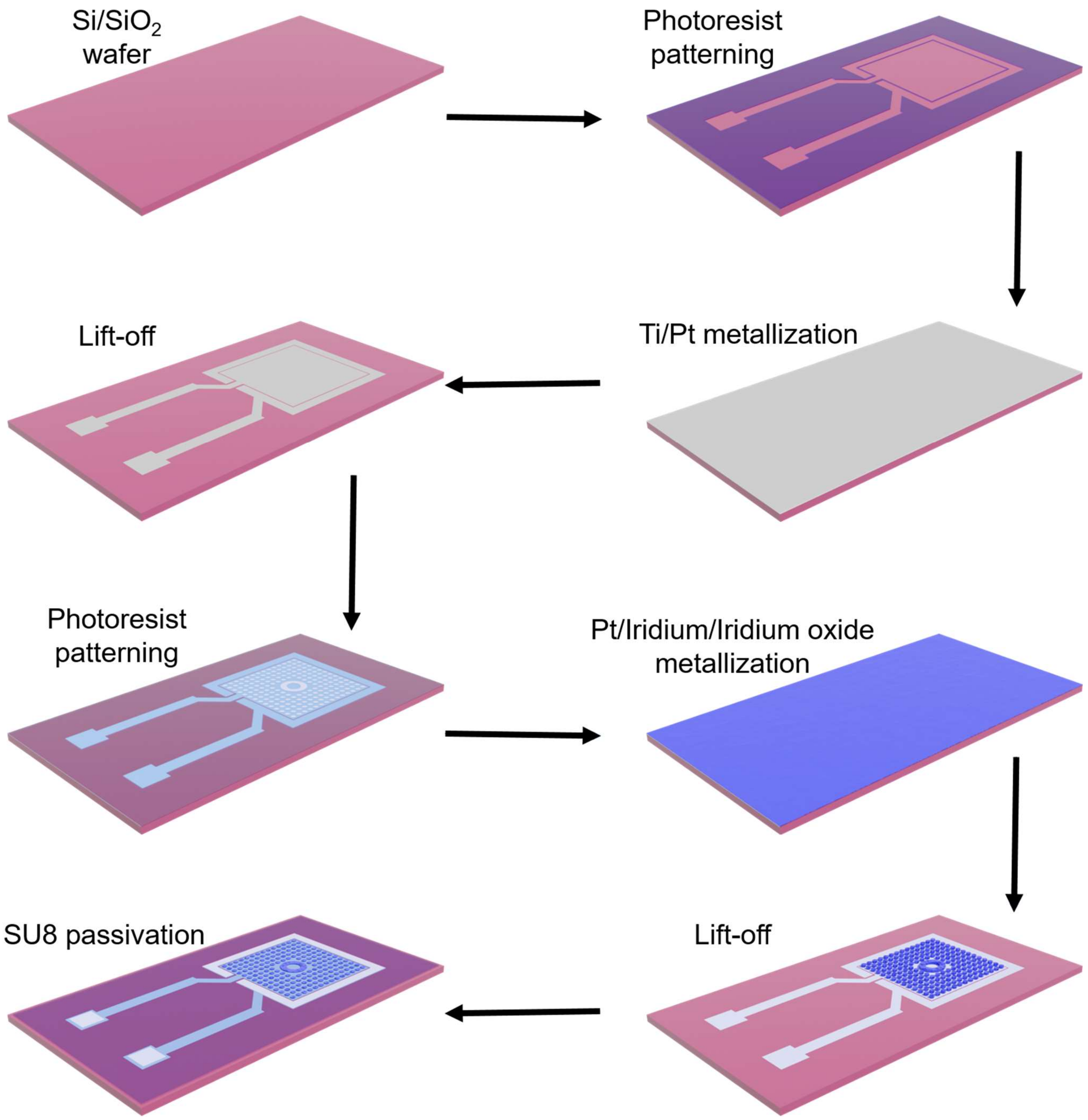

Supplementary Figure 2. ecO_2 microelectrode fabrication scheme. ecO_2 microelectrodes were fabricated on a silicon wafer with 600 nm thick thermally grown SiO_2. Detailed description is provided in materials and methods.

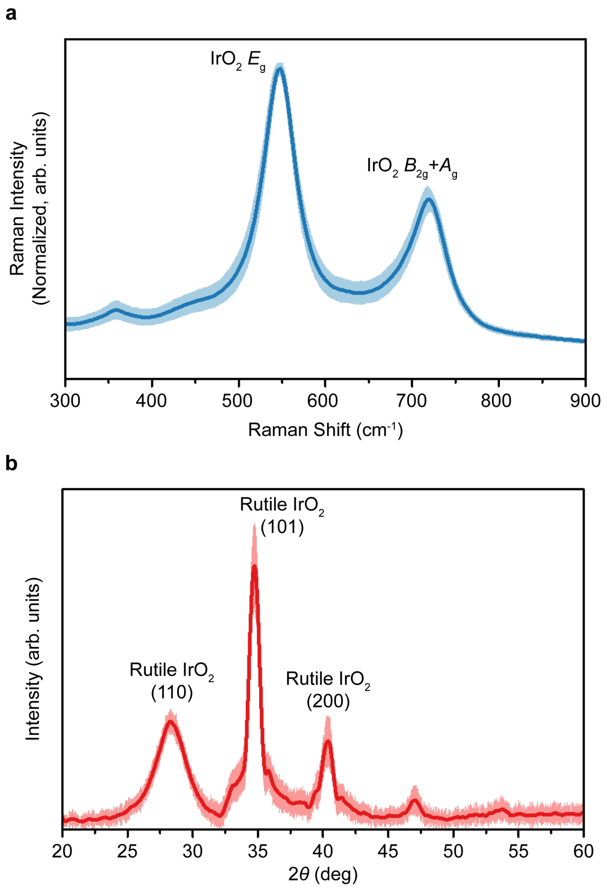

Supplementary Figure 3. Structural analysis of the SIROF catalyst. (a) Raman spectroscopy (blue) and (b) Grazing incidence X-ray diffractometry (GIXRD) (red). Results are presented as mean ± SD (n=6 independent samples for Raman, n=4 independent samples for GIXRD).

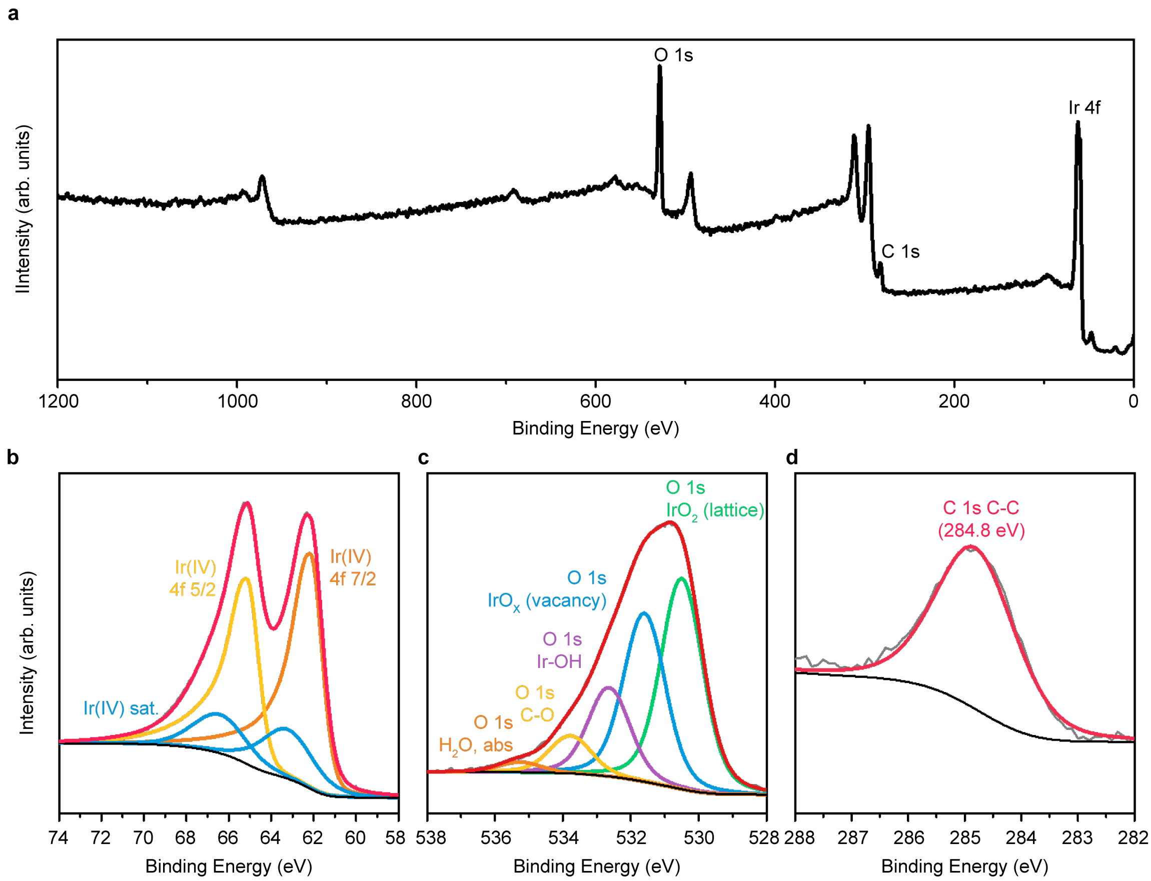

Supplementary Figure 4. X-ray photoemission spectroscopy (XPS) analysis of SIROF. Representative XPS deconvolution; the measurement was repeated in 4 independent samples (a) survey scan; step size – 1 eV (b) Ir 4f; asymmetric doublet curve fitting for Ir(IV) was applied (c) O 1s; (d) C 1s; C-C peak at 284.8 eV was utilized for chemical shift calibration (n=4); step size for high-resolution scan – 0.1 eV.

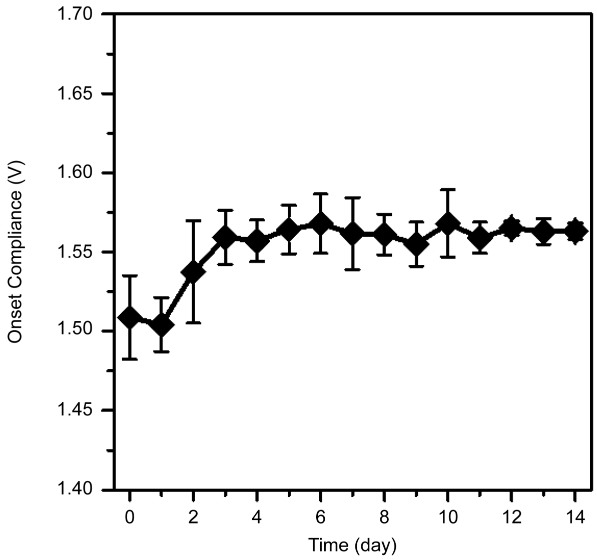

Supplementary Figure 5. ecO_2 onset compliance. Onset values were calculated from 2 electrodes LSV curves which were collected by every 24 hours over 14 days during chronoamperometry at 1.7 V in 1X PBS. Results are represented as mean ± SD (n=5 independent devices)

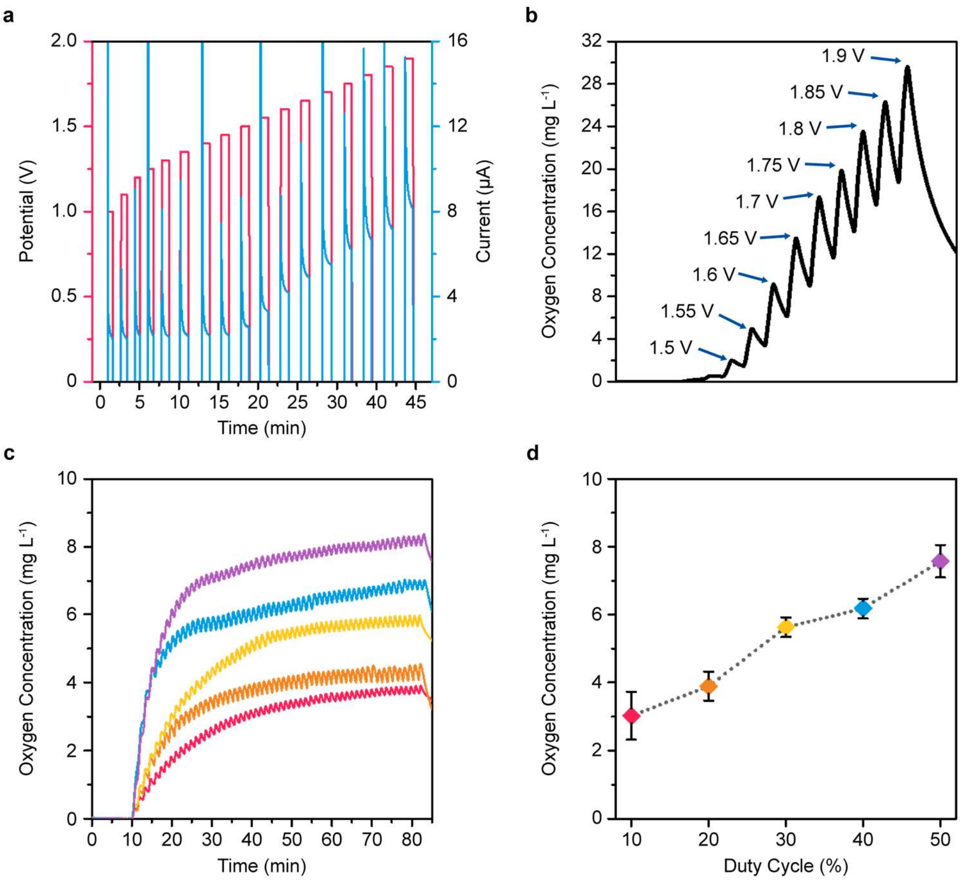

Supplementary Figure 6. Potentiometric electrochemical oxygen generation. (a) A representative recorded current profile (blue) from applied potential (red). (b) A representative measured oxygen concentration profiles with 30-sec potential pulses. (c) A representative produced oxygen profiles by various duty cycles; 10 % (red), 20 % (yellow), 30 % (green), 40 % (blue) and 50 % (purple) at the compliance of 1.7 V. (d) measured oxygen concentration after 60 min oxygen production with varied duty cycles. Results are presented as mean ± SD (n=3 independent devices).

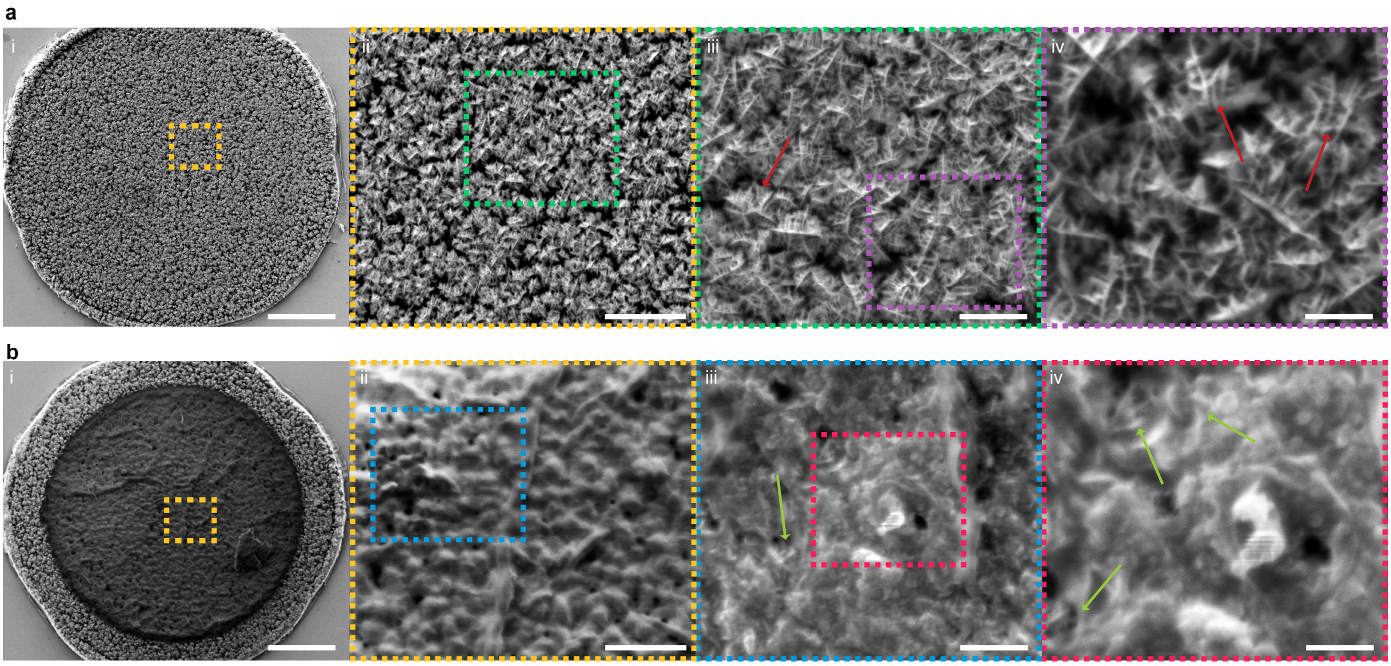

Supplementary Figure 7. ecO_2 before and after 21-day oxygen evolution reaction. Representative SEM images (a) before and (b) after electrochemical oxygen evolution for 21 days with 100 % duty cycle load. The arrows indicated degraded dendritic structures (red: before, green: after); scale bars: i – 10 µm; ii – 2 µm; iii – 1 µm; iv – 500 nm. All images were collected at an accelerating voltage of 1 kV with a working distance of 5 mm. Imaging was repeated in 10 random spots in 3 independent devices.

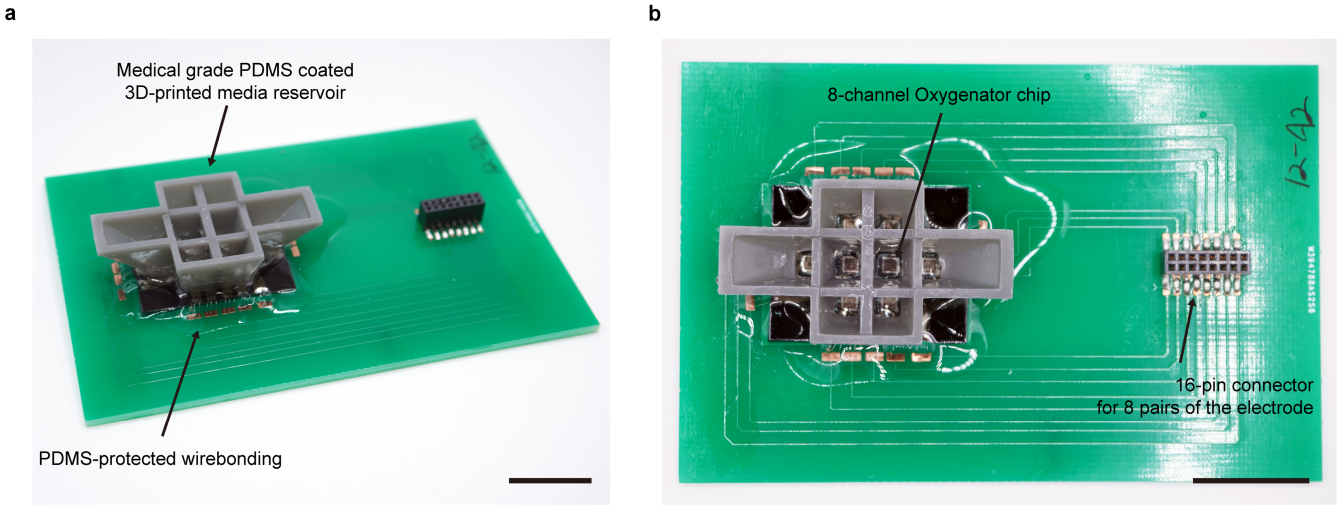

Supplementary Figure 8. ecO_2 in vitro device. Representative images of (a) side view and (b) top view of the in vitro ecO_2. Scale bars are 2 cm.

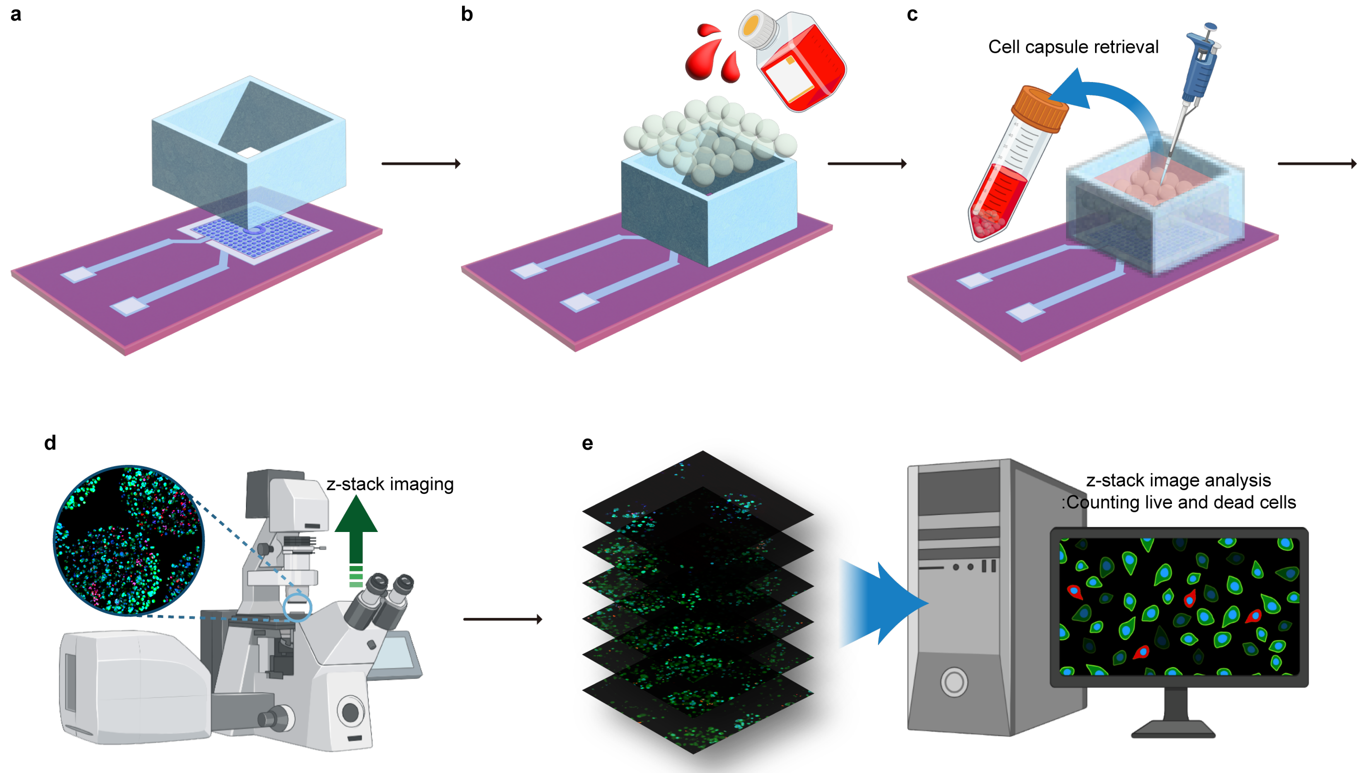

Supplementary Figure 9. A schematic illustration of live/dead assay after in vitro and in vivo oxygenation with ecO_2. (a) Device assembly – media reservoir was attached on microfabricated ecO_2 chip. Note that PDMS encapsulation and immune protection membrane were additionally applied for implantable devices. (b) Cell transfer – alginate cell capsules containing ARPE-19 cells were transferred into the media reservoir with the media. (c) Cell retrieval – after oxygenation, the capsules were collected and calcein/ethidium/hoechst stained to visualize nuclei, live and dead cells. For details, see Materials and Methods. (d) Cell imaging – the stained cells were z-stack imaged using fluorescence confocal microscopy. (e) Image analysis – the recorded z-stack images were analyzed in terms of viability.

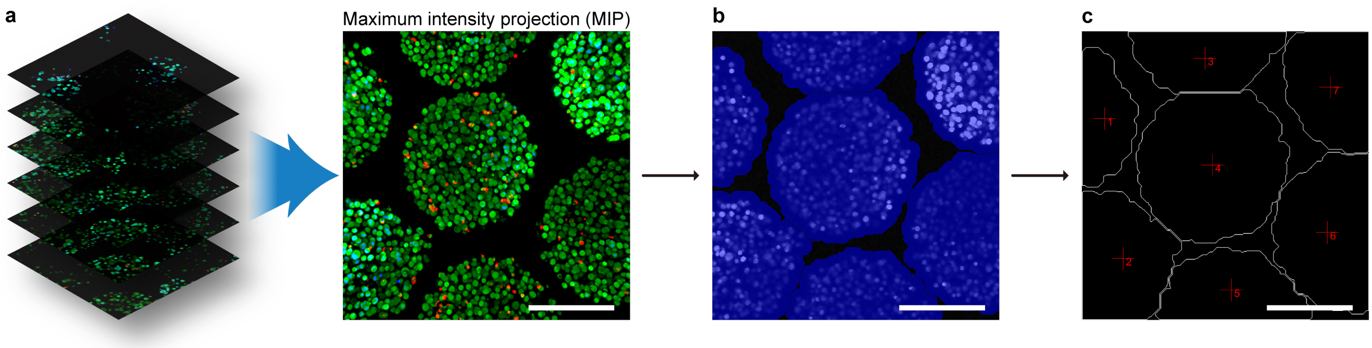

Supplementary Figure 10. Mask creating for single-capsule-level viability assay. (a) MIP image creation – z-stack images were projected in the xy-plane using maximum intensity projection (MIP). (b) Defining capsule footprint – the footprint of each capsule was defined using a watershed of binarized MIP images. (c) Labeling capsules – the defined capsules were labeled; scale bars are 200 µm.

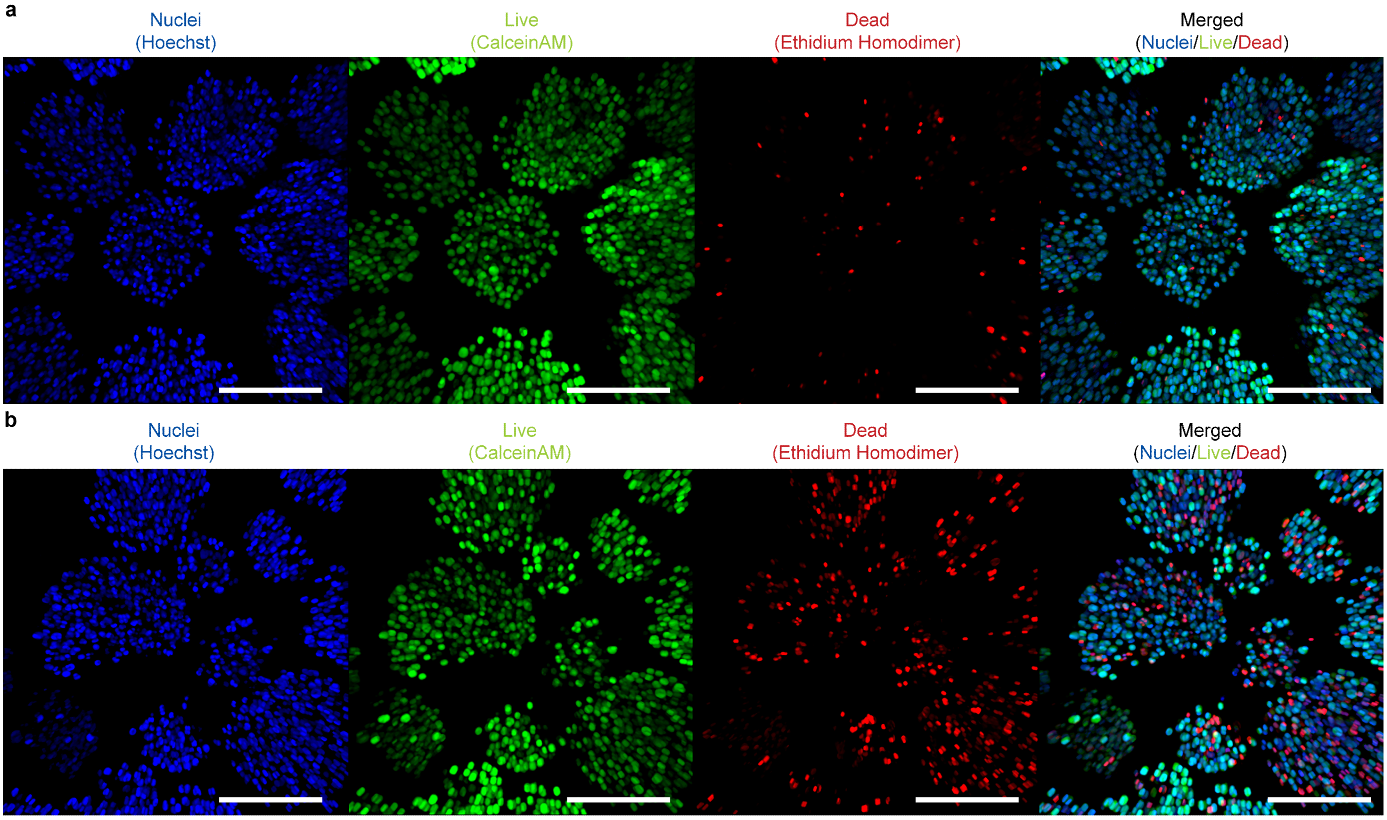

Supplementary Figure 11. Live/dead assay fluorescence images from 3-day in vitro. Representative image for (a) with and (b) without oxygenation. All scale bars are corresponding to 200 µm. Green: CalceinAM (Live), Red: Ethidium homodimer (Dead), Blue: Hoechst (Nuclei); Imaging was repeated in 20 random spots in 4 independent devices.

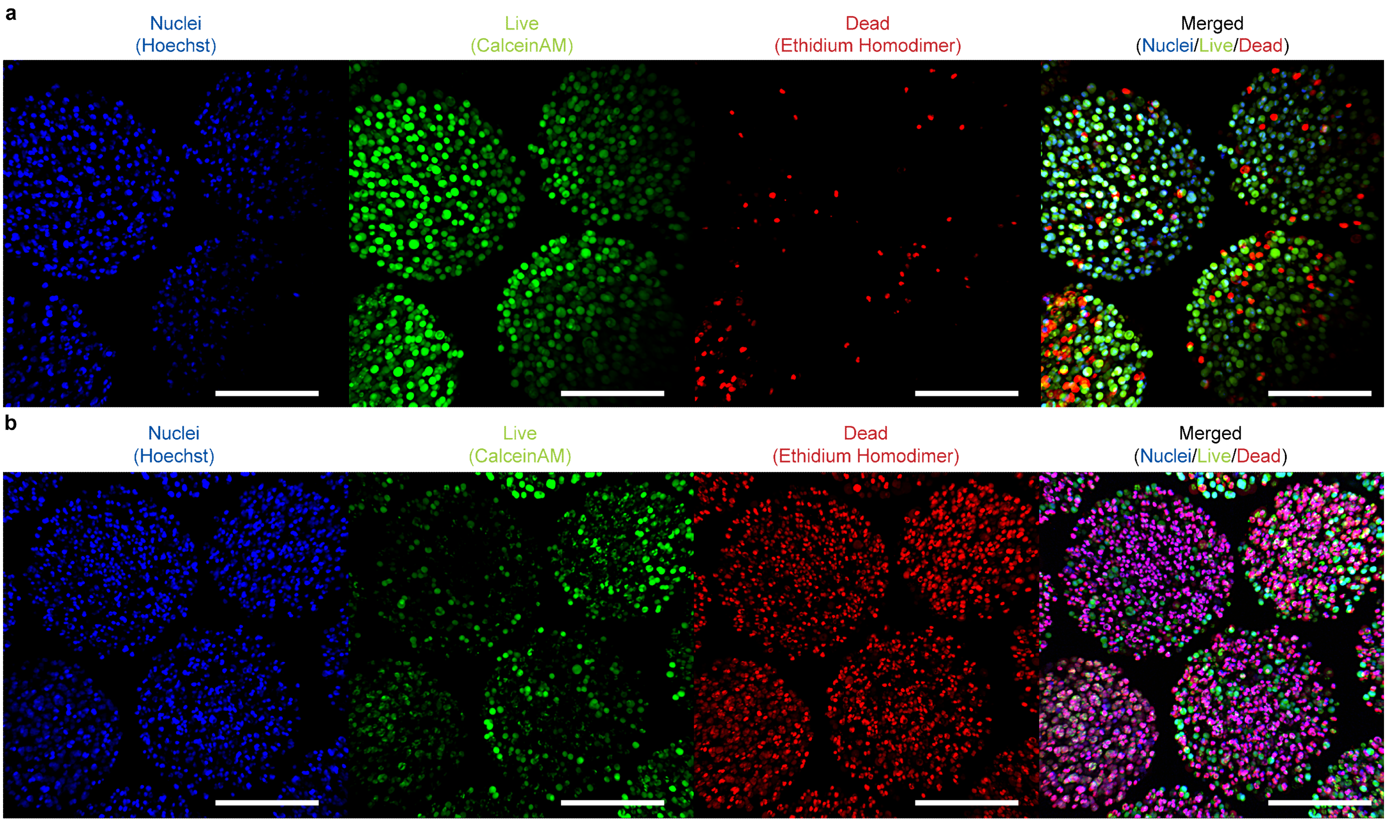

Supplementary Figure 12. Live/dead assay fluorescence images from 10-day in vitro. Representative image for (a) with and (b) without oxygenation. All scale bars are corresponding to 200 μm. Green: CalceinAM (Live), Red: Ethidium homodimer (Dead), Blue: Hoechst (Nuclei); Imaging was repeated in 20 random spots in 4 independent devices.

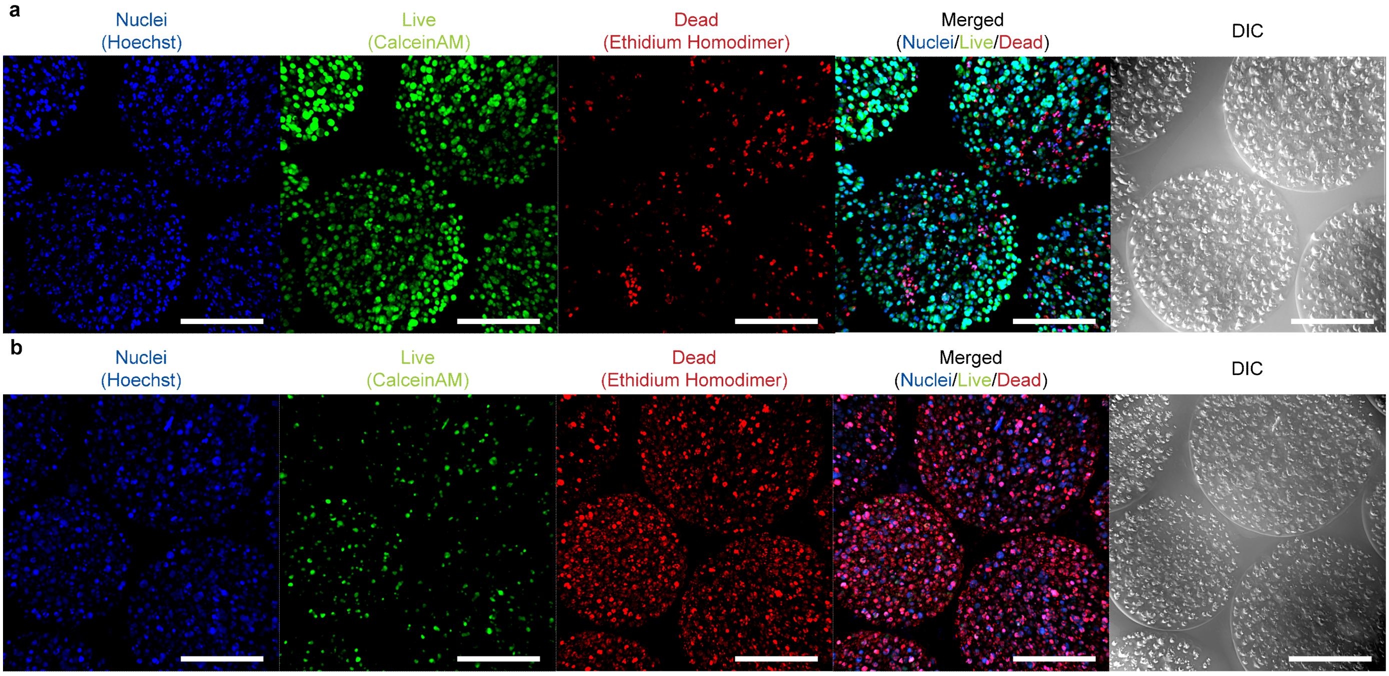

Supplementary Figure 13. Live/dead assay fluorescence images from 21-day in vitro. Representative image for (a) with and (b) without oxygenation. All scale bars are corresponding to 200 µm. Green: CalceinAM (Live), Red: Ethidium homodimer (Dead), Blue: Hoechst (Nuclei), DIC: differential interference contrast images (Cell capsule); Imaging was repeated in 20 random spots in 4 independent samples

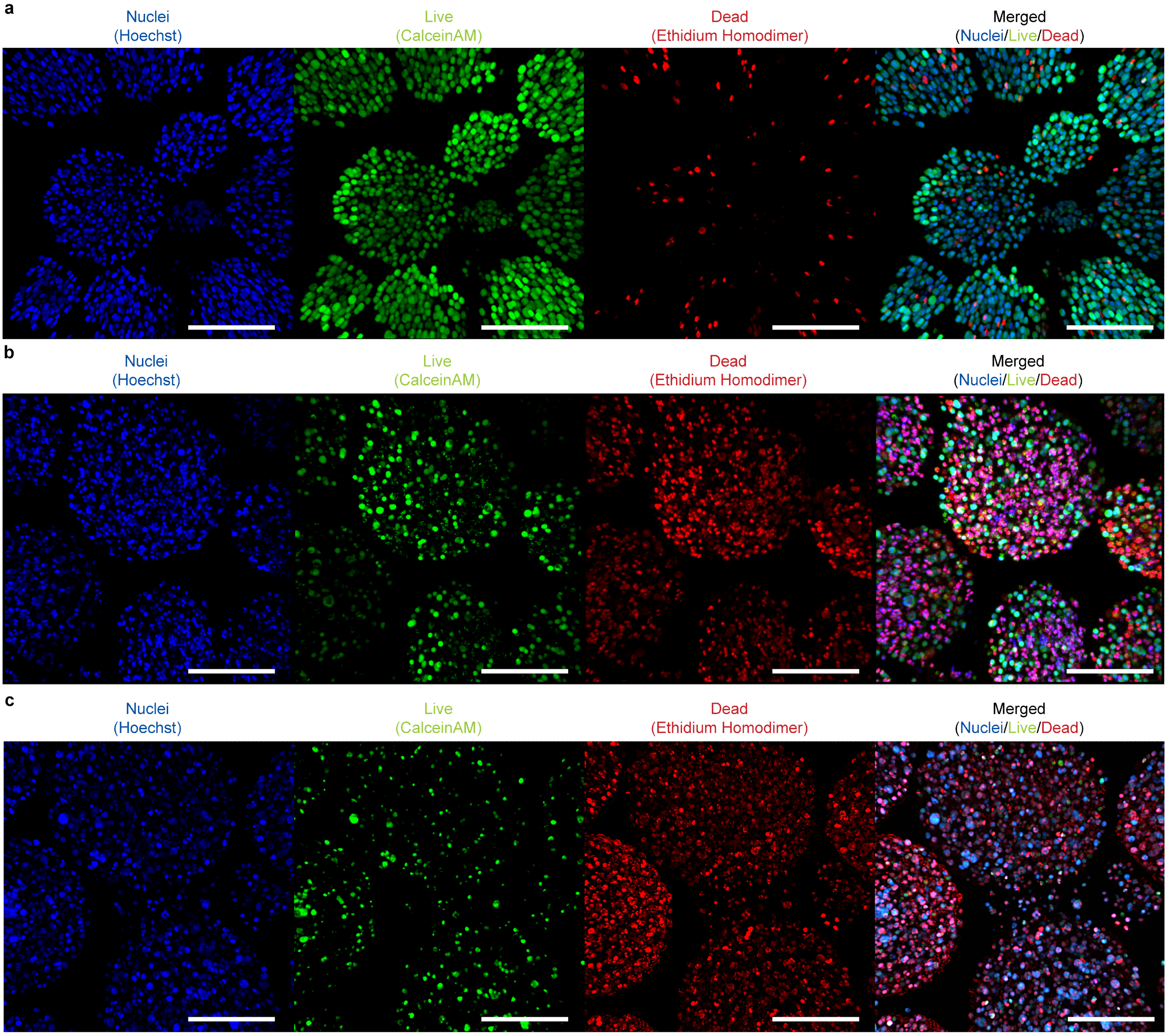

Supplementary Figure 14. Live/dead assay fluorescence images from normoxic incubation (20% O_2) without oxygenation. Representative images for (a) 3, (b) 10 and (c) 21 days. All scale bars are corresponding to 200 µm. Green: CalceinAM (Live), Red: Ethidium homodimer (Dead), Blue: Hoechst (Nuclei); Imaging was repeated in 20 random spots in 4 independent samples.

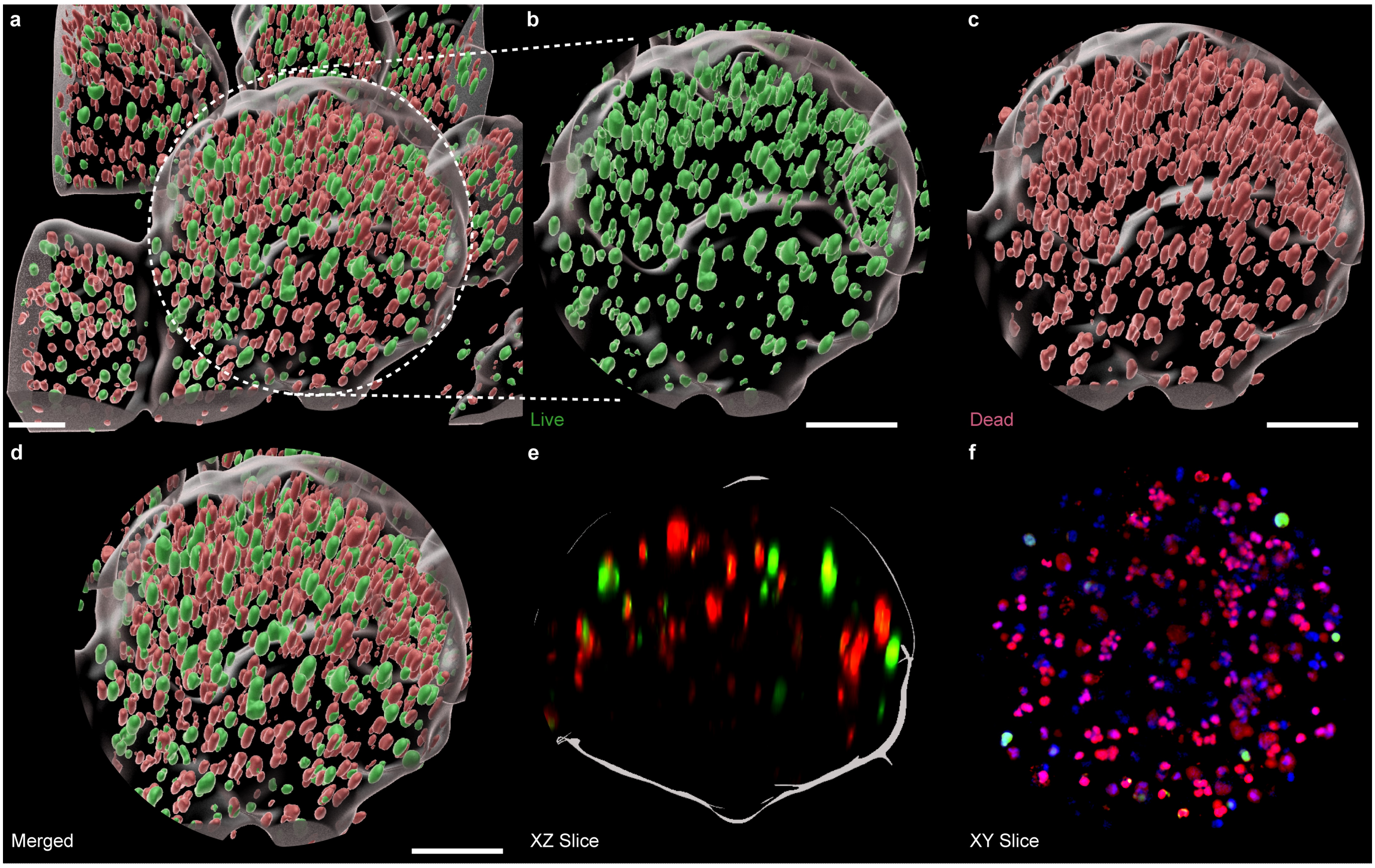

Supplementary Figure 15. 3D-rendered fluorescence images of cell capsules after 21-day normoxic incubation. (a) 3D-reconstructed z-stack live/dead fluorescence images, (b) Live and (c) Dead cells in the white dashes circle marked capsule. A cross-section view in (d) XZ plane and in (e) XY plane. Scale bars are 100 µm. Green: CalceinAM (Live), Red: Ethidium homodimer (Dead), Blue: Hoechst (Nuclei).

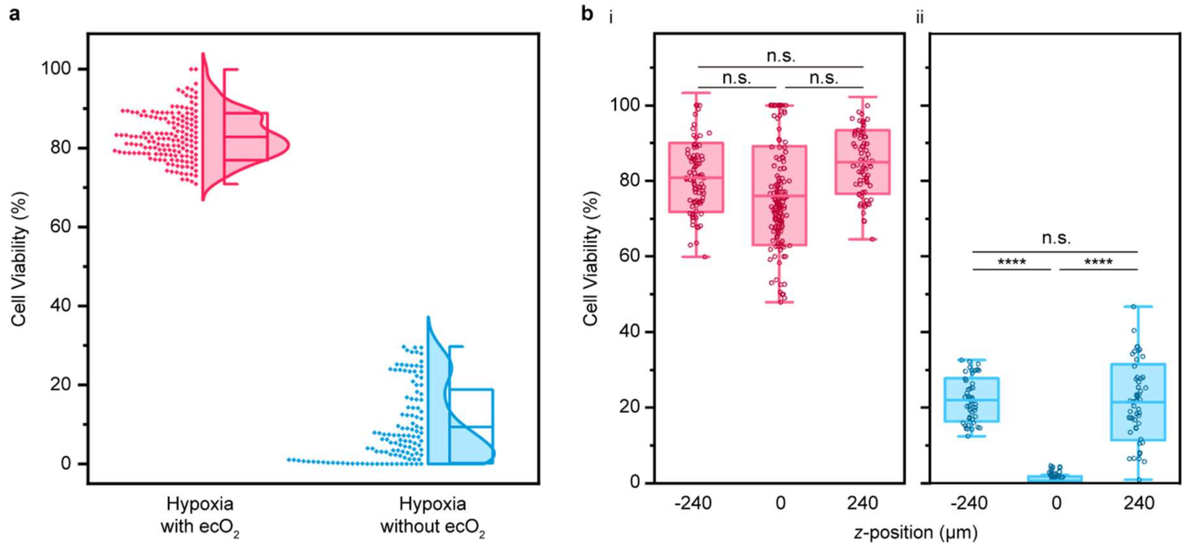

Supplementary Figure 16. Single-capsule-level viability assay of 21-day in vitro oxygenation. (a) Half-violin plots for the viability of individual capsules; red – hypoxia with ecO_2 (n=158 independent cell capsules); blue – hypoxia without ecO_2 (n=122 independent cell capsules); box – standard deviation ; bar – mean; whisker with caps – 1.5 IQR (Interquartile range) (b) statistical analysis of viability at edges (top: 240 μm, bottom: -240 μm) and the center of capsules (center: 0 μm); n.s.: no significance; ****: $p < 0.0001$; $p_{-240-0}=1.77\times10^{-29}$; $p_{240-0}=2.74\times10^{-20}$; results are presented with mean±SD; box – standard deviation; bar – mean; whisker with caps – 1.5 IQR; One-way ANOVA and Tukey post-hoc analysis was performed at the level of (i) $p < 0.05$ and (ii) $p < 0.0001$ for one-sided mean comparison.

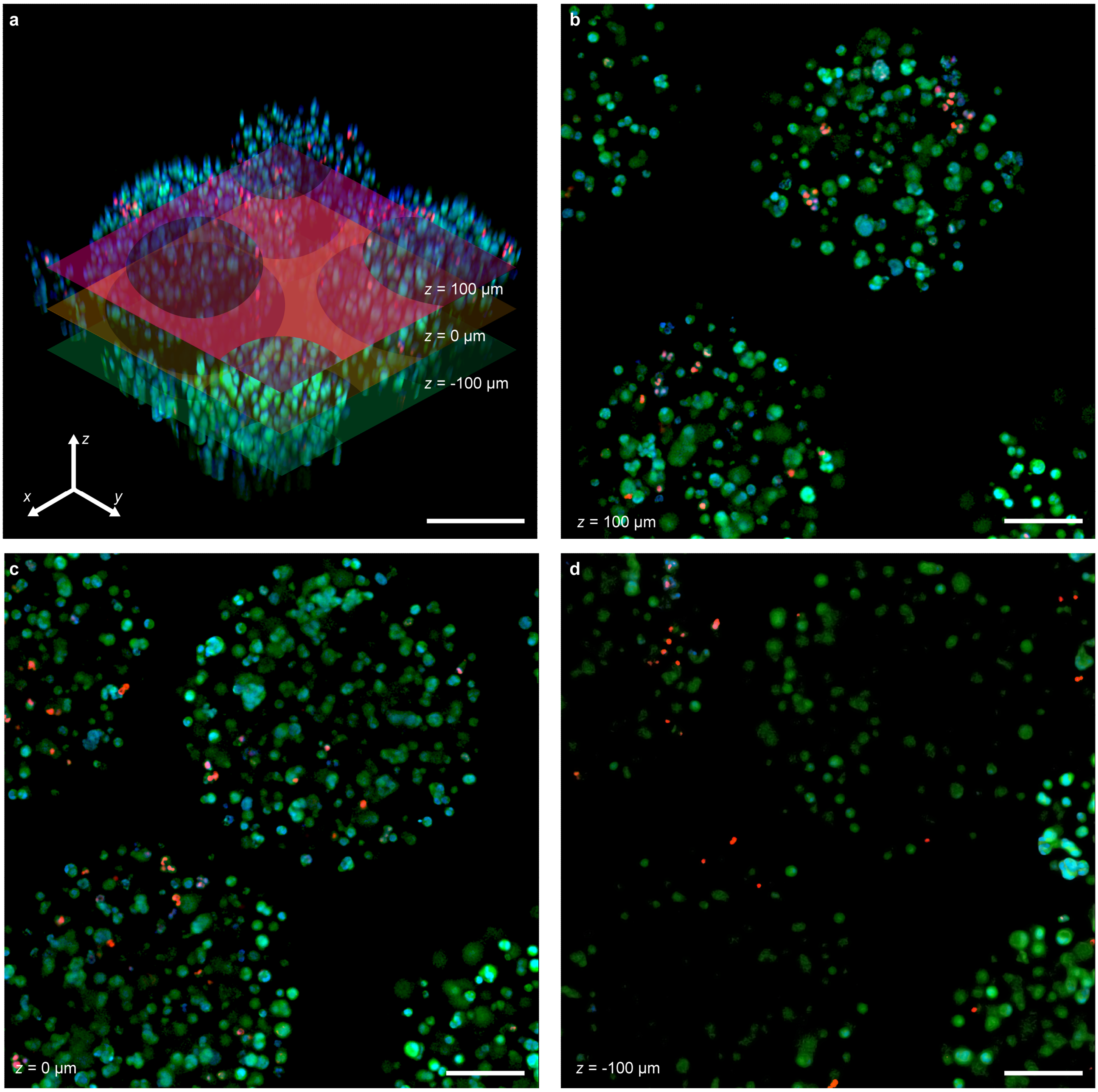

Supplementary Figure 17. Z-stack analysis of 21-day oxygenation. (a) 3D-reconstructed z-stacked images and the location of each presented image. (b) z=100 μm; (c) z=0 μm; (d) z=-100 μm. Scale bars are 100 μm. Green: CalceinAM (Live), Red: Ethidium homodimer (Dead), Blue: Hoechst (Nuclei).

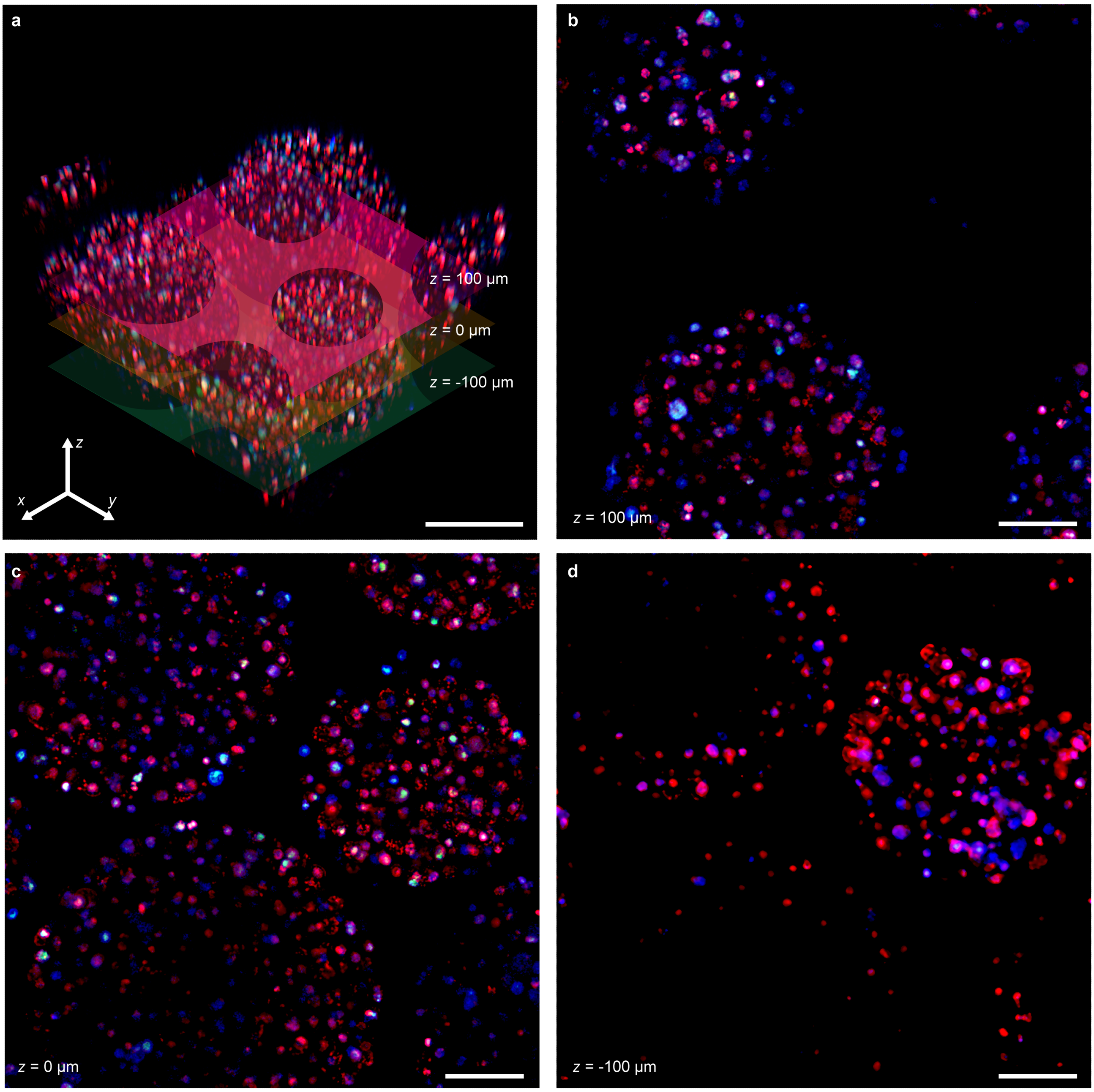

Supplementary Figure 18. Z-stack analysis of 21-day hypoxia (1% O_2) control; (a) 3D-reconstructed z-stacked images and the location of each presented image. (b) z=100 µm; (c) z=0 µm; (d) z=-100 µm; Scale bars are 100 µm. Green: CalceinAM (Live), Red: Ethidium homodimer (Dead), Blue: Hoechst (Nuclei).

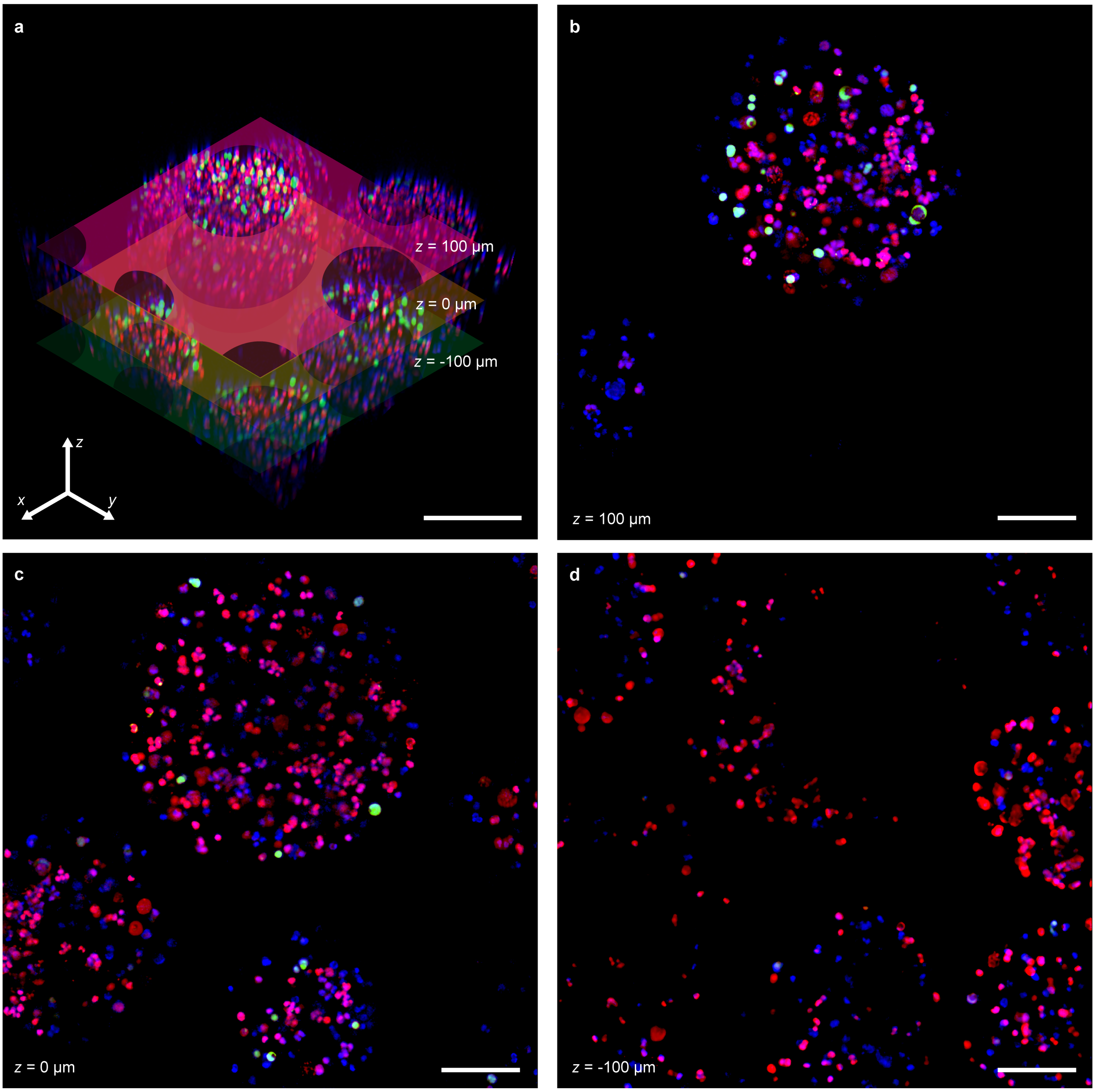

Supplementary Figure 19. z-stack analysis of 21-day normoxia (20% O_2) control; (a) 3D-reconstructed z-stacked images and the location of each presented image. (b) z=100 μm; (c) z=0 μm; (d) z=-100 μm. Scale bars are 100 μm. Green: CalceinAM (Live), Red: Ethidium homodimer (Dead), Blue: Hoechst (Nuclei).

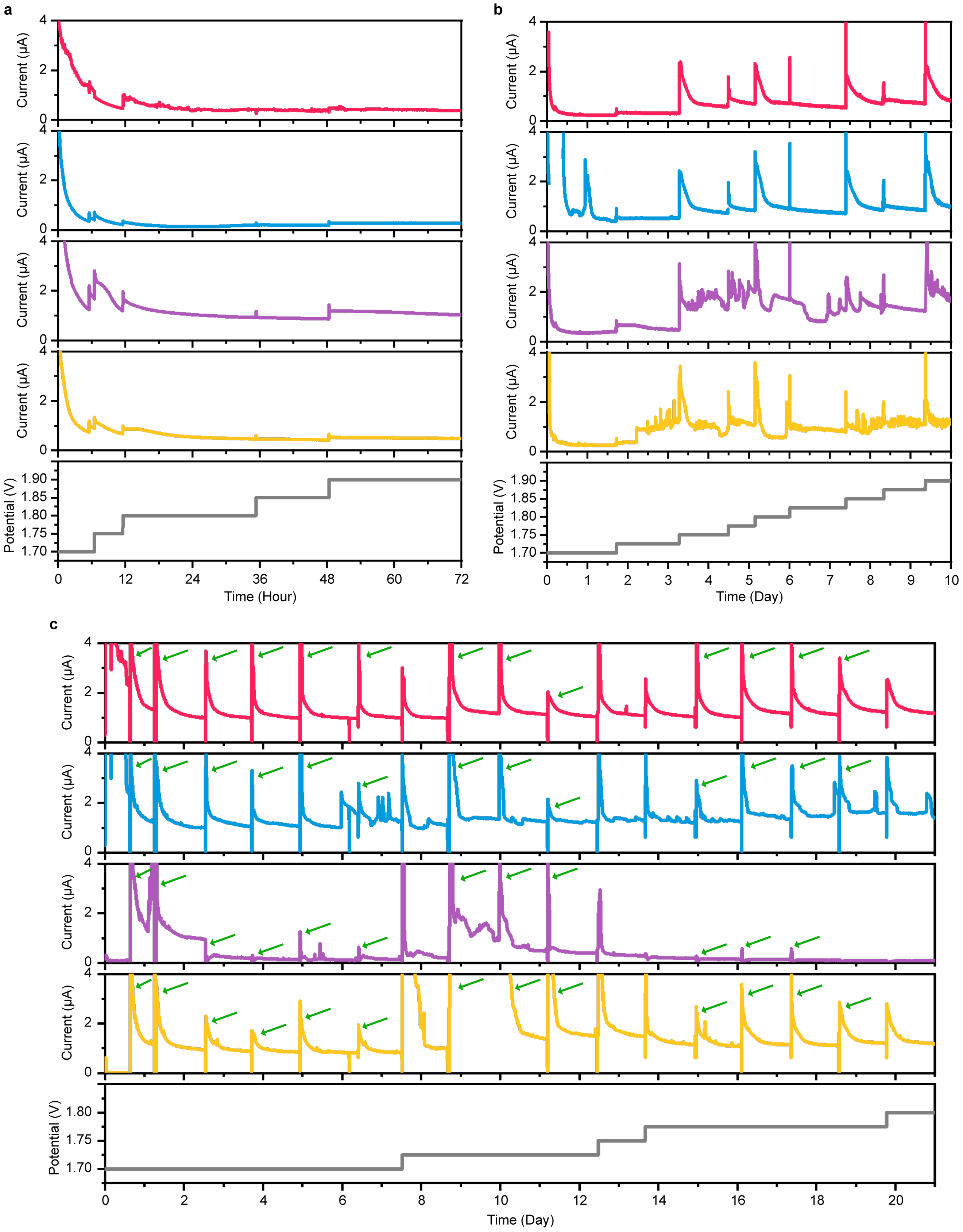

Supplementary Figure 20. Current profiles of in vitro oxygenation. Current profiles from (a) 3-day, (b) 10-day and (c) 21-day in vitro oxygenation; each color corresponding to a device. Note that spikes in the current profiles from 21-day in vitro oxygenation other than potential adjustment are due to media exchange; for example, green arrows indicate current spikes caused by media exchange with deoxygenated media.

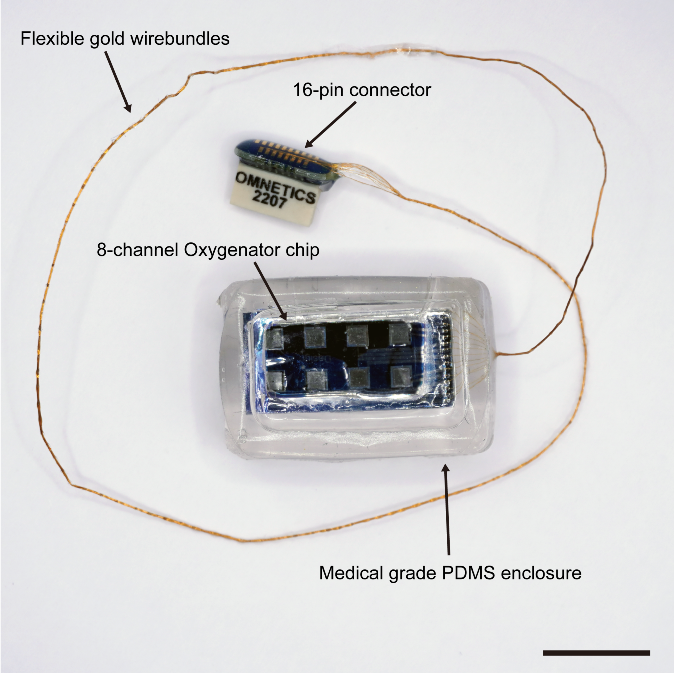

Supplementary Figure 21. ecO_2 in vivo devices. Note that the image was collected without a membrane; scale bar - 1 cm.

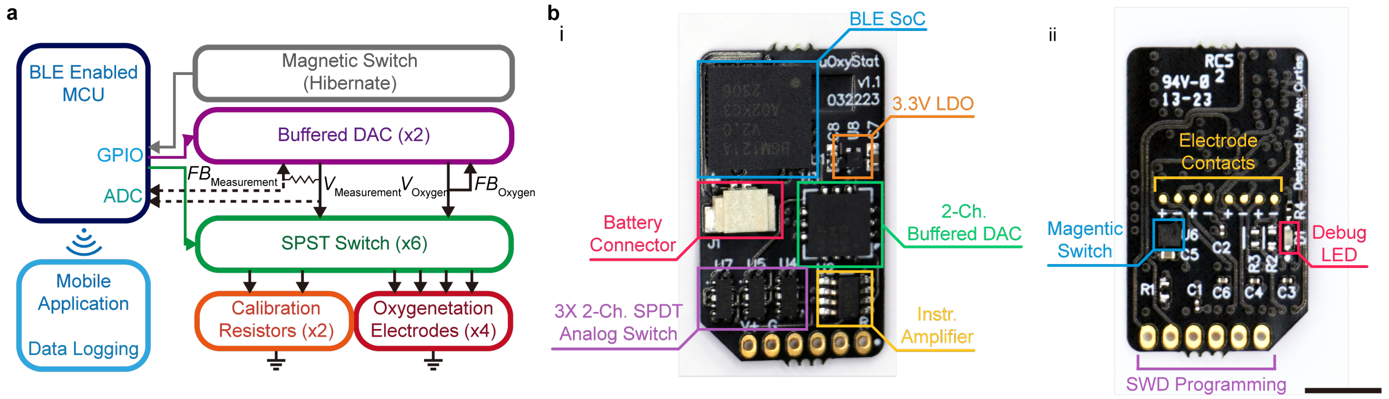

Supplementary Figure 22. Circuit design for in vivo ecO_2. (a) ecO_2 controller circuit design and (b) assembled circuits; (i) front side; (ii) backside. Scale bar is 500 µm.

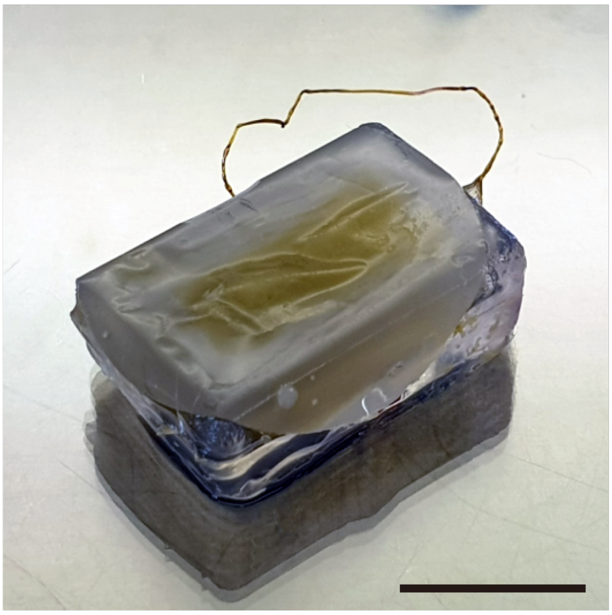

Supplementary Figure 23. ecO_2 post 10 days in vivo. A representative image of retrieved ecO_2 after 10-day. scale bar – 1 cm.

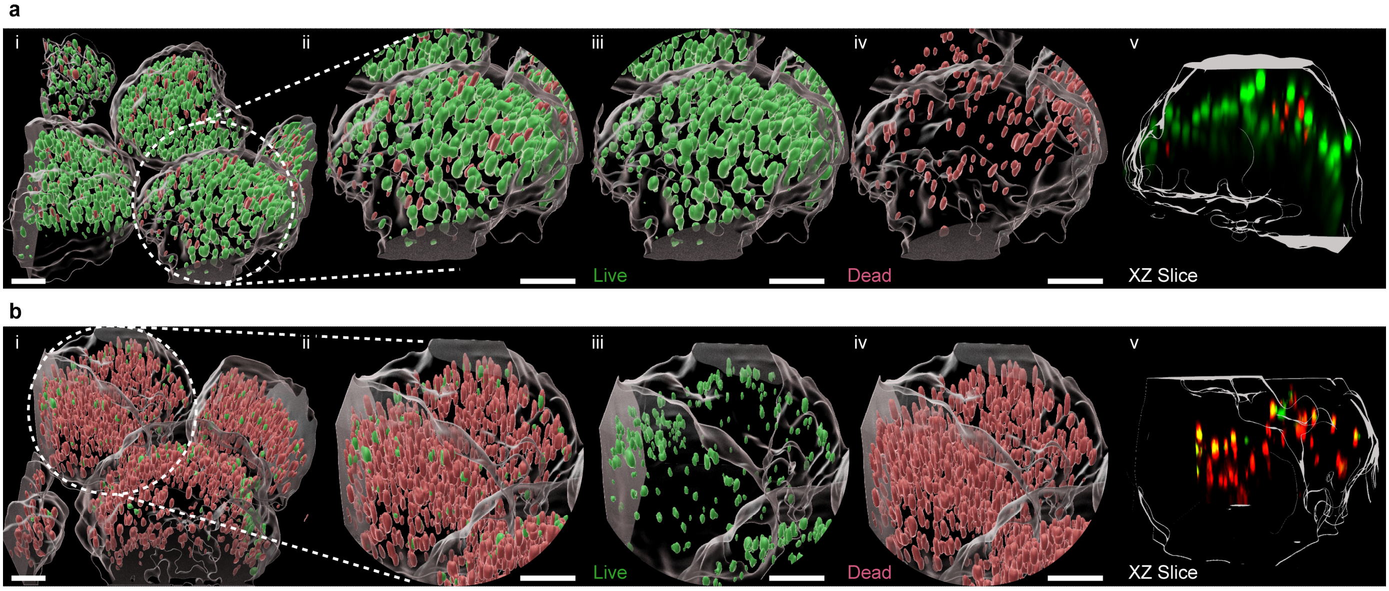

Supplementary Figure 24. 3D-rendered fluorescence images of cell capsules post 10 days in vivo. (a) Representative image of implanted ecO_2 (a) with oxygenation and (b) without oxygenation. (i) 3D-rendered z-stack images. (ii-iv) expanded 3D-rendered images of capsules marked with white dashed circle. (v) Cross-sectional view in xz plane. Scale bars are 100 μm. Green: CalceinAM (Live), Red: Ethidium homodimer (Dead).

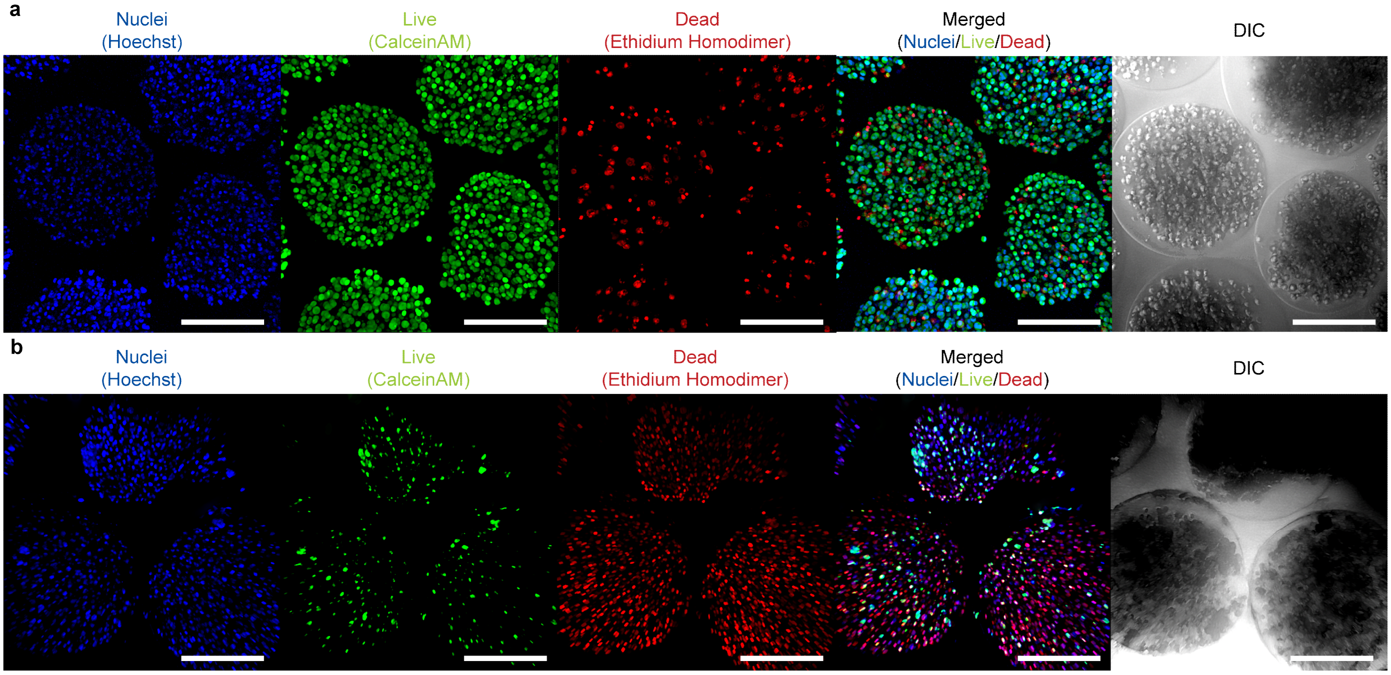

Supplementary Figure 25. Cell viability (live/dead assay) post 10-day in vivo. Representative fluorescence and optical images of capsules (a) with and (b) without oxygenation. Scale bar is 200 μm. Green: CalceinAM (Live), Red: Ethidium homodimer (Dead), Blue: Hoechst (Nuclei), DIC: differential interference contrast images (Cell capsule); Imaging was repeated in 25 random spots in 4 independent devices.

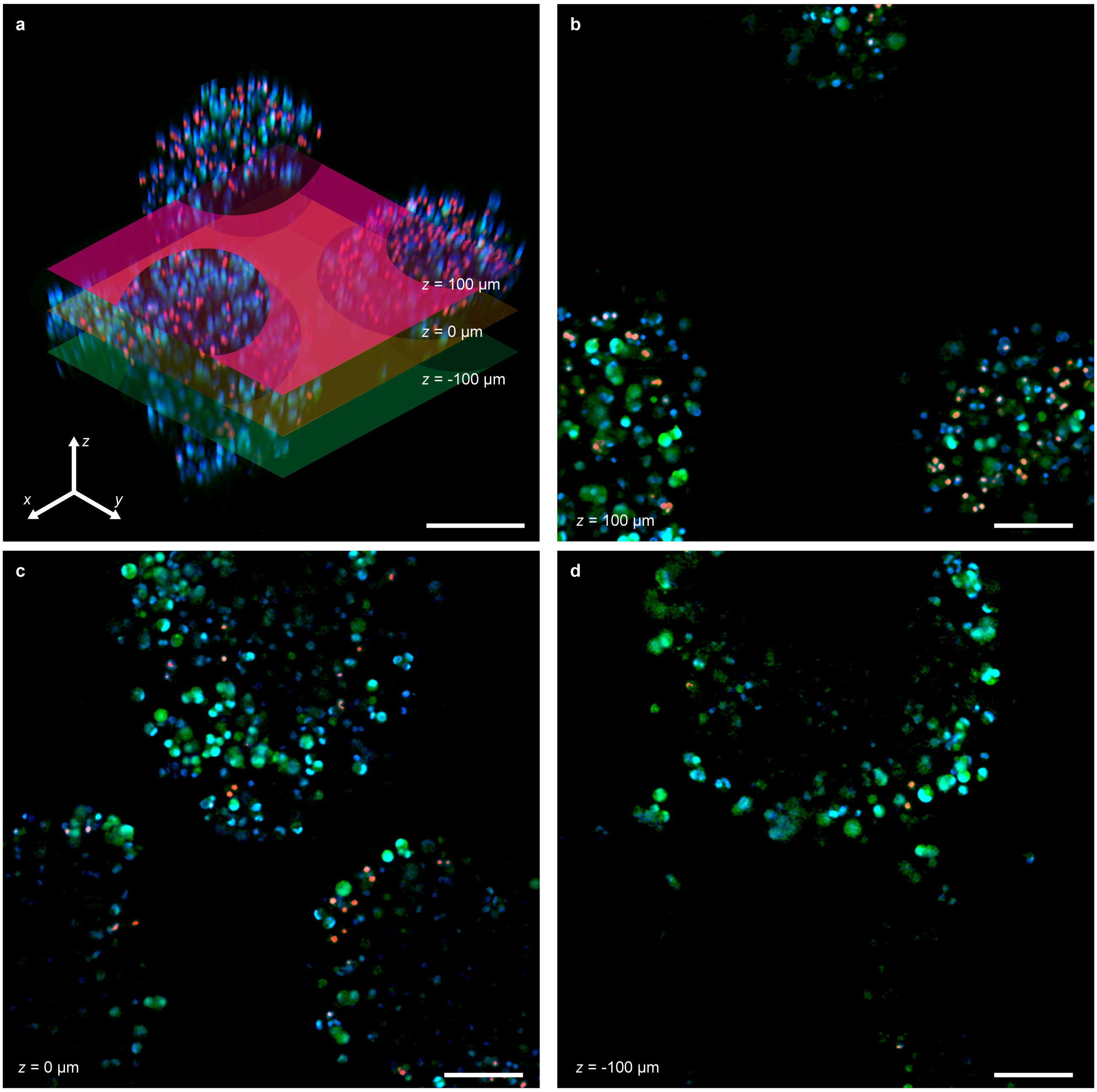

Supplementary Figure 26. z-stack analysis of 10 days in vivo oxygenation; (a) 3D-reconstructed z-stacked images and the location of each presented image. (b) z=100 μm; (c) z=0 μm; (d) z=-100 μm. Scale bars are 100 μm. Green: Calcein (Live), Red: B (Dead), Blue: C (Nuclei).

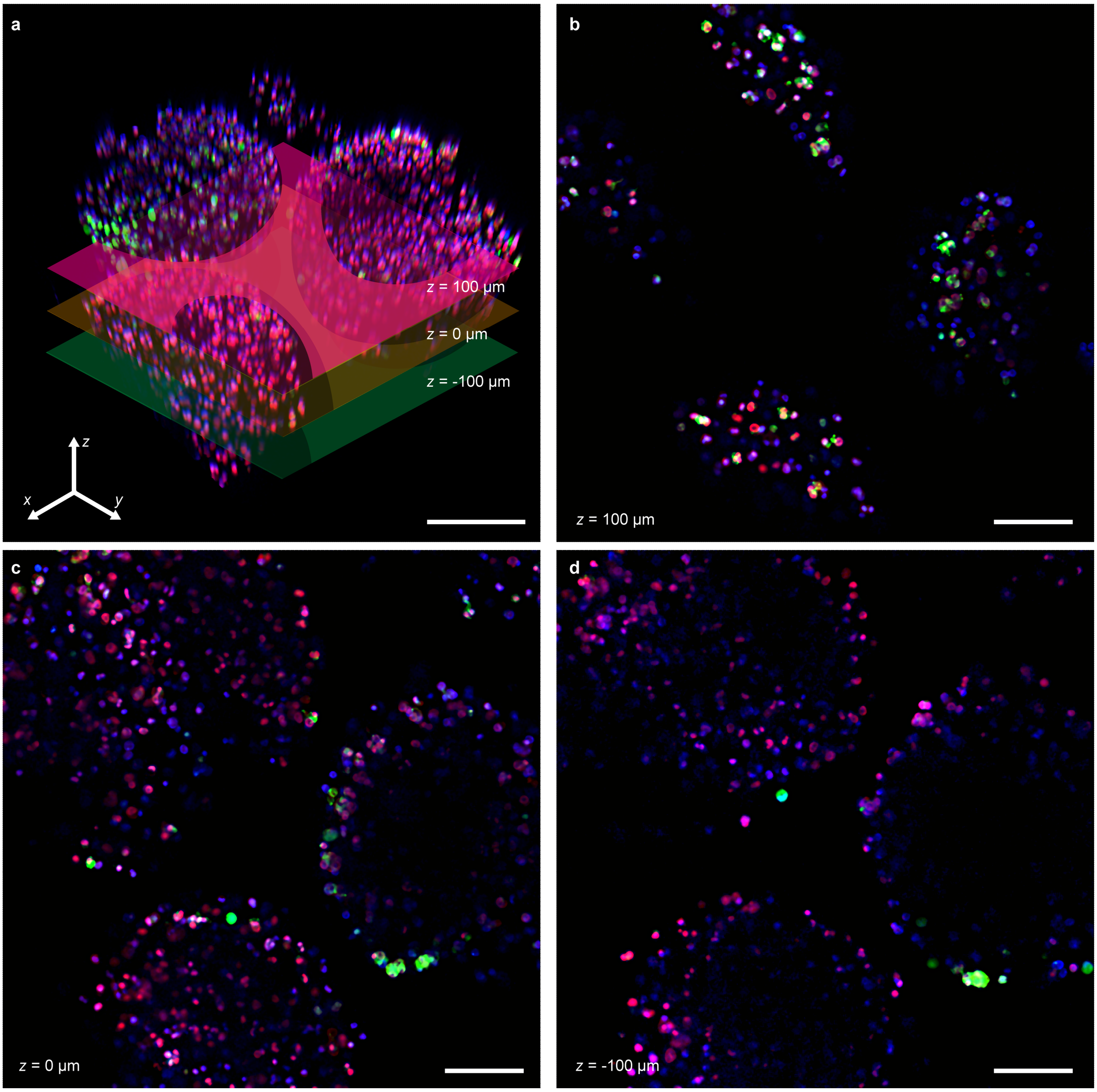

Supplementary Figure 27. z-stack analysis of 10-day in vivo control; (a) 3D-reconstructed z-stacked images and the location of each presented image. (b) z=100 μm; (c) z=0 μm; (d) z=-100 μm. Scale bars are 100 μm. Green: Calcein (Live), Red: B (Dead), Blue: C (Nuclei).

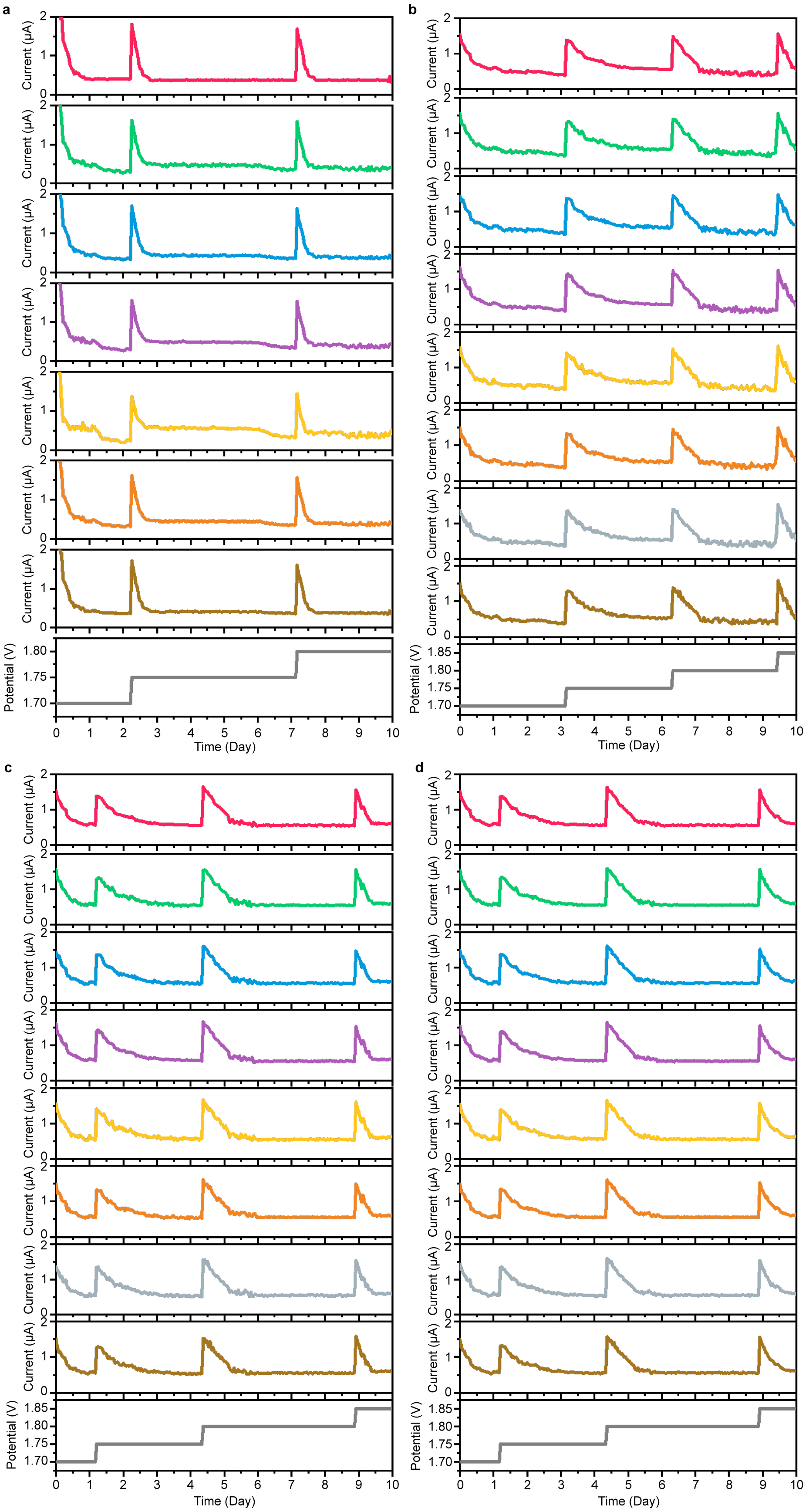

Supplementary Figure 28. Current profiles of in vivo oxygenation. (a-d) Current profiles from each implanted ecO_2 device. All spikes in (b) are corresponding to potential increase, because media exchange was unavailable for the implanted devices. Note that (a) has 7 arrays due to the array failure.

Supplementary Code

Supplementary Code 1. Image analysis code for colorimetric chlorine detection.

```
a = imread('filname.jpg');
b = mean(mean(rgb2gray(a)));
```

Supplementary Code 2. Cell viability evaluation using z-stack fluorescence live/dead images.

```
[file, path, filterindex] = uigetfile('.nd2', 'Select ND2 images', 'MultiSelect',
'on');
    filesno = length(file);
    Image(1:filesno) = struct('B', [], 'G', [], 'R', [],...
                             'Badj', [], 'Gadj', [], 'Radj', [],...
                             'Nuc', [], 'Live', [], 'Dead', [], 'NucNum', [],
'LiveNum', [], 'DeadNum', []);
for i=1:filesno
[WholeImage] = ND2ReadSingle(char(file(i)))
[xpix,ypix,zlayer] = size(WholeImage{1})
for j = 1:zlayer
    Image(i).B = WholeImage{1}(:,:,j);
    Image(i).G = WholeImage{2}(:,:,j);
    Image(i).R = WholeImage{3}(:,:,j);

    Image(i).Badj = wiener2(imadjust(Image(i).B),[10 10]);
    Image(i).Gadj = wiener2(imadjust(Image(i).G),[10 10]);
    Image(i).Radj = wiener2(imadjust(Image(i).R),[10 10]);

    Image(i).Dead= imbinarize(Image(i).Radj, 0.6);
    Image(i).Live= imbinarize(Image(i).Gadj, 0.55);
    Image(i).Nuc= imbinarize(Image(i).Badj, 0.5);

    Image(i).Dead= bwareaopen(Image(i).Dead,20);
    Image(i).Live= bwareaopen(Image(i).Live,35)
    Image(i).Nuc= bwareaopen(Image(i).Nuc,35);

    [DeadLabel, Image(i).DeadNum(j)] = bwlabel(Image(i).Dead);

    [LiveLabel, Image(i).LiveNum(j)] = bwlabel(Image(i).Live);
    se = strel('disk',1);
    a = imdilate(Image(i).Live, se);
    D = bwdist(~a);
    D = -D;
    mask=imextendedmin(D,0.5);
    D2=imimposemin(D,mask);
    Ld2=watershed(D2);
    bw3=a;
    bw3(Ld2==0)=0;
    Image(i).NumWaterLC = bw3
    [LiveLabel, Image(i).LiveNum(j)] = bwlabel(bw3);

    se = strel('disk',1);
    b = imdilate(Image(i).Nuc, se);
    E = bwdist(~b);
    E = -E;
    mask=imextendedmin(E,0.5);
    E2=imimposemin(E,mask);
    Ld3=watershed(E2);
    bw4=b;
    bw4(Ld3==0)=0;
    Image(i).NumWater = bw4
    [NucLabel, Image(i).NucNum(j)] = bwlabel(bw4);
end

end
```

Supplementary Code 3. Single-capsule masking using maximum intensity projection (MIP) image.

```
%% INITIATION

Blue = 1;
Green = 2;
Red = 3;
DIC = 4;

IntensityAll = [];
CellNumber = [];
AreaAll = [];

[file, path, filterindex] = uigetfile('.nd2', 'Select ND2 images', 'MultiSelect',
'on');
    filesno = length(file);
    Image(1:filesno) = struct('Blue', [], 'Green', [], 'Red', [], 'DIC', [],...
                              'Badj', [], 'Gadj', [], 'Radj', [],...
                              'Nuc', [], 'Live', [], 'Dead', [],...
                              'NucNum', [], 'LiveNum', [], 'DeadNum', []);

for i = 1:filesno
    [WholeImage] = ND2ReadSingle(char(file(i)));
    [rows, columns, numSlices] = size(WholeImage{1});

for j = 1:numSlices
    Image(i).Blue(:, :, j) = WholeImage{Blue}(:, :, j);
    Image(i).Green(:, :, j) = WholeImage{Green}(:, :, j);
    Image(i).Red(:, :, j) = WholeImage{Red}(:, :, j);
end

end

%% MAXIMUM INTENSITY PROJECTION (MIP)

for i = 1:filesno
 [rows, columns, numSlices] = size(Image(i).Blue);
outputImage(i).Blue = zeros(rows, columns);
for col = 1:columns
    for row = 1:rows
        Zvector(i).Blue = Image(i).Blue(row, col, :);
        maxValue(i).Blue = max(Zvector(i).Blue);
        outputImage(i).Blue(row, col) = maxValue(i).Blue;
    end

end

end

for i = 1:filesno
    [rows, columns, numSlices] = size(Image(i).Green);
    outputImage(i).Green = zeros(rows, columns);
for col = 1:columns
    for row = 1:rows
        Zvector(i).Green = Image(i).Green(row, col, :);
        maxValue(i).Green = max(Zvector(i).Green);
        outputImage(i).Green(row, col) = maxValue(i).Green;
    end
```

```matlab
end

end

for i = 1:filesno
    [rows, columns, numSlices] = size(Image(i).Red);
    outputImage(i).Red = zeros(rows, columns);
for col = 1:columns
    for row = 1:rows
        Zvector(i).Red = Image(i).Red(row, col, :);
        maxValue(i).Red = max(Zvector(i).Red);
        outputImage(i).Red(row, col) = maxValue(i).Red;
    end

end

end

for i = 1:filesno
    outputImage(i).MIP = outputImage(i).Blue + outputImage(i).Green +
outputImage(i).Red;
    imshow(outputImage(i).MIP);

end

%% IMAGE SEGMENTATION WITH MIP IMAGES

for i = 1:filesno
    Normalization(i) = max(outputImage(i).MIP, [], 'all');
    MIPnorm{i} = outputImage(i).MIP/Normalization(i);
    imshow(MIPnorm{i});
    title('raw image(i)');

    MIPadj{i} = wiener2(imadjust(MIPnorm{i}), [5 ,5]);
    imshow(MIPadj{i});
    title('Adjusted original image(i)')

    MIPbi{i} = imbinarize(MIPadj{i}, 0.125);
    imshow(MIPbi{i});
    title('binary MIP(i)');

    MIPNR{i} = bwareaopen(MIPbi{i}, 10);
    imshow(MIPNR{i});
    title('Filtered MIP(i)');

    se = strel('line', 7, 7);
    MIPdi{i} = imdilate(MIPNR{i}, se);
    imshow(MIPdi{i});
    title('Dilated MIP(i)');

    MIPfill{i} = imfill(MIPdi{i}, 'holes');
    imshow(MIPfill{i});
    title('Gap-filled MIP(i)');

    se = strel("disk",30);
    a = imdilate(MIPfill{i}, se);
    D = bwdist(~a);
```

```
    D = -D;
    mask=imextendedmin(D,20);
    D2=imimposemin(D,mask);
    Ld2=watershed(D2);
    MIPWS{i}=a;
    MIPWS{i}(Ld2==0)=0;
    imshow(MIPWS{i});
    title('Watershed');

    MIPcaps{i} = bwareaopen(MIPWS{i}, 1000);
    imshow(MIPcaps{i});
    title('Filtered integral capsule(i)');

MIPlabeled{i} = bwlabel(MIPcaps{i});
CapsMeasured{i} = regionprops(MIPlabeled{i},'Area','Centroid');

allArea = [CapsMeasured{i}.Area]';
allCentroid = [CapsMeasured{i}.Centroid];

imshow(labeloverlay(MIPadj{i}, MIPcaps{i}))
print([file{i},'CellMaskOverLay.png'],'-dpng','-r600');

MIPCapsEdge{i} = bwperim(MIPcaps{i});
imshow(MIPCapsEdge{i});
hold on;
for k = 1:length(CapsMeasured{i})
    plot(allCentroid(2*k - 1),allCentroid(2*k),'r+', 'MarkerSize', 30);
    hold on
    text(allCentroid(2*k -
1)+5,allCentroid(2*k)+5,num2str(k),'Color','red','FontSize',14);
    hold on;
end
hold off;
title('Cell Mask - number');
print([file{i},'CellNum.png'],'-dpng','-r600');

close all

end
```
