# Supplementary material for: Electrocatalytic on-site oxygenation for transplanted cell-based-therapies
Source: Nat Commun. 2023 Nov 9;14:7019. doi: 10.1038/s41467-023-42697-2 (PMC10636048; doi:10.1038/s41467-023-42697-2)
Supplement: Supplementary file 3 — Reporting Summary [file 41467_2023_42697_MOESM3_ESM.pdf]

nature portfolio

Corresponding author(s): Tzahi Cohen-Karni, Jonathan Rivnay

Last updated by author(s): Oct 4, 2023

Reporting Summary

Nature Portfolio wishes to improve the reproducibility of the work that we publish. This form provides structure for consistency and transparency in reporting. For further information on Nature Portfolio policies, see our Editorial Policies and the Editorial Policy Checklist.

Statistics

For all statistical analyses, confirm that the following items are present in the figure legend, table legend, main text, or Methods section.

n/a	Confirmed	
☐	☒	The exact sample size (*n*) for each experimental group/condition, given as a discrete number and unit of measurement
☐	☒	A statement on whether measurements were taken from distinct samples or whether the same sample was measured repeatedly
☐	☒	The statistical test(s) used AND whether they are one- or two-sided<br>*Only common tests should be described solely by name; describe more complex techniques in the Methods section.*
☒	☐	A description of all covariates tested
☒	☐	A description of any assumptions or corrections, such as tests of normality and adjustment for multiple comparisons
☐	☒	A full description of the statistical parameters including central tendency (e.g. means) or other basic estimates (e.g. regression coefficient) AND variation (e.g. standard deviation) or associated estimates of uncertainty (e.g. confidence intervals)
☐	☒	For null hypothesis testing, the test statistic (e.g. *F*, *t*, *r*) with confidence intervals, effect sizes, degrees of freedom and *P* value noted<br>*Give P values as exact values whenever suitable.*
☒	☐	For Bayesian analysis, information on the choice of priors and Markov chain Monte Carlo settings
☒	☐	For hierarchical and complex designs, identification of the appropriate level for tests and full reporting of outcomes
☒	☐	Estimates of effect sizes (e.g. Cohen's *d*, Pearson's *r*), indicating how they were calculated

Our web collection on statistics for biologists contains articles on many of the points above.

Software and code

Policy information about availability of computer code

Data collection	Electrochemical data were acquired using the equipment provided software (Gamry Analyst, PalmSens PSTrace 5.9). Optical images including bright field and fluorescence images were collected using Nikon NIS Element software. Scanning electron microscopy was performed using XT Microscope Control and Server accompanied with FEI Quanta 600 FEG Microscope. Well-plate reading for hydrogen peroxide quantification was done using BioTek Gen5 software. Quantification of dissolved oxygen concentration was performed using PreSens Measurement Studio.
Data analysis	All data plotting and statistical analyses in the manuscript were done using OriginPro 2021. Image analyses for cell viability evaluation were performed utilizing ImageJ and custom Matlab codes (MatLab R2020b) that include binarization and watershed to count cells.

For manuscripts utilizing custom algorithms or software that are central to the research but not yet described in published literature, software must be made available to editors and reviewers. We strongly encourage code deposition in a community repository (e.g. GitHub). See the Nature Portfolio guidelines for submitting code & software for further information.

Data

Policy information about availability of data

All manuscripts must include a data availability statement. This statement should provide the following information, where applicable:

- Accession codes, unique identifiers, or web links for publicly available datasets
- A description of any restrictions on data availability
- For clinical datasets or third party data, please ensure that the statement adheres to our policy

All relevant datasets supporting key findings in this study within the manuscript and the Supplementary Information, or from the corresponding authors upon

reasonable requests. Source data is provided with the article.

Research involving human participants, their data, or biological material

Policy information about studies with human participants or human data. See also policy information about sex, gender (identity/presentation), and sexual orientation and race, ethnicity and racism.

Reporting on sex and gender	N/A
Reporting on race, ethnicity, or other socially relevant groupings	N/A
Population characteristics	N/A
Recruitment	N/A
Ethics oversight	N/A

Note that full information on the approval of the study protocol must also be provided in the manuscript.

Field-specific reporting

Please select the one below that is the best fit for your research. If you are not sure, read the appropriate sections before making your selection.

[x] Life sciences [ ] Behavioural & social sciences [ ] Ecological, evolutionary & environmental sciences

For a reference copy of the document with all sections, see nature.com/documents/nr-reporting-summary-flat.pdf

Life sciences study design

All studies must disclose on these points even when the disclosure is negative.

Sample size	For statistical analysis, we predetermined effective sample size based on power analysis, executing all in vitro and in vivo experiments with biologically independent replications of more than 4.
Data exclusions	Collected data points were analyzed using box plot to exclude outlying data (> 1.5 IQR)
Replication	in vitro Experiments were biollogically quadruplicated and technially triplicated. Animal experiments were biologically quadruplicated. All attempts generated comparable outcomes.
Randomization	The randomization is not relevant to the study because there was only one experimental group with controlled oxygenation in vitro and in vivo.
Blinding	The blinding is not relevant to the study because there was only experimental group with controlled oxygenation in vitro and in vivo. For a rat model, blinding is not necessary, because there were no significant variations in the tested animals.

Reporting for specific materials, systems and methods

We require information from authors about some types of materials, experimental systems and methods used in many studies. Here, indicate whether each material, system or method listed is relevant to your study. If you are not sure if a list item applies to your research, read the appropriate section before selecting a response.

Materials & experimental systems

n/a	Involved in the study	
[x]	[ ]	Antibodies
[ ]	[x]	Eukaryotic cell lines
[x]	[ ]	Palaeontology and archaeology
[ ]	[x]	Animals and other organisms
[x]	[ ]	Clinical data
[x]	[ ]	Dual use research of concern
[x]	[ ]	Plants

Methods

n/a	Involved in the study	
[x]	[ ]	ChIP-seq
[x]	[ ]	Flow cytometry
[x]	[ ]	MRI-based neuroimaging

Eukaryotic cell lines

Policy information about cell lines and Sex and Gender in Research

Cell line source(s)	The parental ARPE-19 cell line used was purchased from ATCC. Engineered ARPE-19 were created from this parental line with a piggybac transpose system.
Authentication	Parental cell line was authenticated by the vendor ATCC using STR profiling.
Mycoplasma contamination	All cell lines were tested and found to be negative for mycoplasma using R&D MycoProbe Mycoplasma Detection kit.
Commonly misidentified lines (See ICLAC register)	None

Animals and other research organisms

Policy information about studies involving animals; ARRIVE guidelines recommended for reporting animal research, and Sex and Gender in Research

Laboratory animals	Rat, SAS Sprague Dawley, 8 weeks old
Wild animals	No wild animal were used in this study.
Reporting on sex	Male and female rats were mixed
Field-collected samples	No field collected samples were used in this study
Ethics oversight	All procedures were approved by the Carnegie Mellon University Institutional Animal Care and Use Committee and the DoD Animal Care and Use Review Office.

Note that full information on the approval of the study protocol must also be provided in the manuscript.
